# The NLRP3 Inflammasome in Neuropsychiatric Disorders: Molecular Mechanisms and Emerging Therapeutic Strategies

**DOI:** 10.3390/ijms27073127

**Published:** 2026-03-30

**Authors:** Monica Neamțu, Tudor Petreuș, Doinița Temelie Olinici, Laura Stoica, Oana Dana Arcan, Bogdan Alexandru Stoica, Corneliu Moșoiu

**Affiliations:** 1 Department of Physiology, Cell and Molecular Biology, Faculty of Medicine, Grigore T. Popa University of Medicine and Pharmacy Iasi, 700115 Iasi, Romania; monica.neamtu@umfiasi.ro (M.N.); doinita.p.olinici@umfiasi.ro (D.T.O.); laura.stoica@umfiasi.ro (L.S.); 2 Department of Pharmacodynamics and Clinical Pharmacy, Faculty of Pharmacy, Grigore T. Popa University of Medicine and Pharmacy Iasi, 700115 Iasi, Romania; oana-dana.arcan@umfiasi.ro; 3 Department of Biochemistry, Faculty of Medicine, Grigore T. Popa University of Medicine and Pharmacy Iasi, 700115 Iasi, Romania; bogdan.stoica@umfiasi.ro; 4 Department of Psychology, Faculty of Social and Human Sciences, Lucian Blaga University of Sibiu, 550024 Sibiu, Romania; corneliu.mosoiu@ulbsibiu.ro

**Keywords:** inflammasome, neuroinflammation, neuropsychiatric disorders, inflammasome targets

## Abstract

Inflammasomes are cytosolic multiprotein complexes that detect pathogens, cellular stress, and damage-associated molecular signals, thereby orchestrating innate immune responses. Increasing evidence suggests that dysregulated inflammasome activation contributes to persistent neuroinflammation and to a wide range of neuropsychiatric disorders, including mood disorders, schizophrenia, Alzheimer’s disease, and autism spectrum disorders. Together, these findings emphasize the critical role of neuroimmune interactions in the pathophysiology of mental disorders. Recent molecular studies have substantially advanced our understanding of the crosstalk among neurons, microglia, astrocytes, and peripheral immune cells, uncovering complex regulatory networks mediated by cytokines, neurotrophins, and neurotransmitters. By examining key inflammatory mediators and cell type-specific mechanisms, this review consolidates current knowledge and proposes conceptual frameworks to guide future investigations and facilitate the development of targeted therapeutic strategies for neuropsychiatric disorders.

## 1. Introduction

### 1.1. Neuroinflammation

Neuropsychiatric disorders comprise a heterogeneous group of conditions associated with abnormalities in the limbic system, including the thalamus, hypothalamus, hippocampus, and amygdala, as well as in the cerebral cortex [1]. Chronic stress is a major driver of neuroimmune imbalance and contributes to the emergence of inflammatory processes within the central nervous system (CNS) [2].

Neuroinflammation is a complex, multidimensional process involving dynamic interactions among multiple cellular and molecular components. Under physiological conditions, this tightly regulated crosstalk supports central nervous system adaptability and resilience; however, it becomes dysregulated during neuroinflammatory states. Microglia, astrocytes, neurons, peripheral immune cells, and major signaling mediators, including cytokines, neurotransmitters, extracellular matrix (ECM) components, and neurotrophic factors, represent the principal elements of neuroimmune communication [3]. This review provides a mechanistic, integrative, cross-disorder perspective centered on the NLRP3 (nucleotide-binding oligomerization domain-like receptor family pyrin domain-containing 3) inflammasome as a convergent neuroimmune hub linking mitochondrial dysfunction, barrier pathology, glial activation, and cytokine signaling across neuropsychiatric and neurodegenerative disorders. Therapeutic implications are discussed as a translational extension of this framework.

### 1.2. Neuroinflammation Mechanism

Neuroinflammation encompasses a range of inflammatory processes that arise within the central nervous system under pathological conditions. These processes are governed by complex and dynamic interactions among multiple brain cell types and signaling mediators, including cytokines, neurotrophins, and neurotransmitters, which collectively modulate brain function, immune activity, and inflammatory responses [4]. Sustained exposure to stressors triggers molecular and cellular cascades that promote long-term neurobiological alterations, thereby increasing vulnerability to psychiatric conditions such as depression, post-traumatic stress disorder (PTSD), and anxiety disorders [5].

In parallel, neuroinflammation is increasingly recognized as a key contributor to the pathogenesis of neurodegenerative disorders, including Alzheimer’s disease, Parkinson’s disease, Huntington’s disease, and multiple sclerosis. It is also associated with age-related cognitive decline and dementia, both of which involve progressive memory loss and neuronal dysfunction [6,7].

## 2. Inflammasomes

Inflammasomes are cytosolic multiprotein complexes that detect pathogens, cellular stress, and damage-associated molecular patterns (DAMPs). Several inflammasomes have been identified, including AIM2 (absent in melanoma 2), NLRC4 (NLR family CARD domain-containing protein 4), and NLRP1 (NLR family pyrin domain-containing protein 1), yet NLRP3 remains the most extensively studied. These complexes are central components of innate immunity, acting as molecular scaffolds that assemble in response to pathogen-associated molecular patterns (PAMPs) and DAMPs and regulate inflammatory signaling at the post-translational level [8]. Although inflammasome activation is essential for host defense, aberrant activation and gain-of-function mutations affecting inflammasome-related genes have been linked to the development and progression of autoimmune and autoinflammatory diseases [9].

Because inflammasomes are implicated in a wide spectrum of inflammatory and autoimmune conditions, they have become major targets of translational research. In particular, increasing attention has focused on their therapeutic modulation as a potential anti-inflammatory strategy. Upon activation, inflammasome complexes mediate the cleavage of the inactive precursor forms of IL-1β, IL-18, and IL-33 into their biologically active pro-inflammatory forms [10].

Inflammasomes are generally classified into NLR, AIM2-like receptor, and pyrin inflammasome families. Canonical inflammasomes include NLRP1, NLRP3, NLRC4, AIM2, and pyrin, whereas non-canonical inflammasome signaling is mediated by caspase-4, caspase-5, and caspase-11 [11].

Different inflammasomes have different activation mechanisms. For example, NLRP3 can react to a wide range of agonists that are widely available and have distinct structural and chemical characteristics, whereas AIM2 can only be activated by DNA from bacteria or viruses. Because of this, NLRP3 is the most versatile inflammasome with the widest functional range in both the innate and adaptive immune systems [12,13].

### 2.1. Activation and Assembly of Inflammasomes

The NLRP3 inflammasome is activated by a broad array of stimuli, including viruses, bacteria, fungi, pore-forming toxins, crystalline materials such as monosodium urate, silica, asbestos, and alum, and extracellular ATP. Despite their marked structural diversity, these stimuli converge on common intracellular events that promote inflammasome activation [14]. In general, the triggers of innate inflammatory responses are categorized as PAMPs, which originate from infectious agents, and DAMPs, which signal cellular stress or tissue damage. These molecular cues are sensed by pattern recognition receptors (PRRs) expressed predominantly by innate immune cells, thereby initiating inflammatory signaling cascades [15].

Activation of the NLRP3 inflammasome is classically described as a two-step process comprising priming and activation. During the priming phase, Toll-like receptors (TLRs) and other upstream sensors recognize PAMPs and DAMPs, leading to activation of the nuclear factor kappa B (NF-κB) pathway. NF-κB translocates to the nucleus and induces the transcription of NLRP3, ASC, pro-caspase-1, pro-IL-1β, and pro-IL-18 [16].

**Figure 1:**
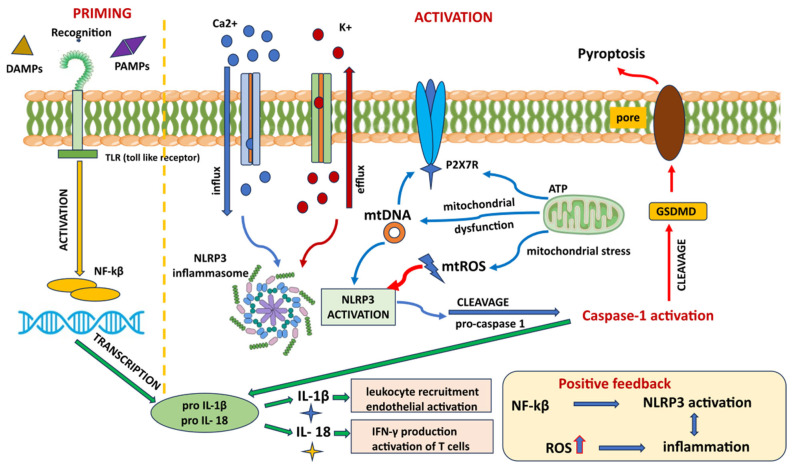
Schematic mechanistic overview of NLRP3 inflammasome activation. DAMPs—damage-associated molecular patterns; GSDMD—gasdermin D; NLRP3—nucleotide-binding domain, leucine-rich repeat family, pyrin domain containing protein 3; mtDNA—mitochondrial DNA; mtROS—mitochondrial reactive oxygen species; NF-KB—nuclear factor kappa B; ROS—reactive oxygen species; P2X7R—purinergic receptor P2X, type 7; IL-1β—interleukin 1β; IL-18—interleukin 18; IFN-γ—interferon gamma.

Subsequent inflammasome assembly is driven by a second signal and is further fine-tuned by a complex network of post-transcriptional and post-translational regulatory mechanisms [17]. Key activating events include potassium efflux through P2X7 receptors, intracellular calcium elevation, endoplasmic reticulum stress, and increased production of reactive oxygen species (ROS). Once activated, the inflammasome promotes caspase-1 autocleavage, resulting in the processing of gasdermin D (GSDMD). The N-terminal fragment of GSDMD forms pores in the plasma membrane, enabling the release of mature IL-1β and IL-18. These cytokines subsequently amplify inflammatory responses and may contribute to the onset and progression of depression [18] (Figure 1).

The priming stage is initiated by cytokines or PAMPs, which induce the transcriptional upregulation of NLRP3 inflammasome components. The activation stage is driven by multiple upstream signals, including K^+^ efflux [17], Ca^2+^ flux [19], and mitochondrial reactive oxygen species (mtROS) generation [20]. Inflammasome assembly promotes caspase-1 activation, leading to the maturation of IL-1β and IL-18. Concurrently, cleavage of gasdermin D (GSDMD) generates an N-terminal fragment that inserts into the plasma membrane, forms pores, and triggers pyroptosis. Positive feedback mechanisms further amplify this pathway: secreted IL-1β activates IL-1 receptor/NF-κB signaling in neighboring cells, increasing pro-IL-1β and NLRP3 expression, while inflammasome-associated cellular stress enhances ROS production and mitochondrial damage, thereby sustaining NLRP3 activation.

Multiple positive feedback loops amplify NLRP3 inflammasome signaling. Secreted IL-1β binds to IL-1 receptors on neighboring cells, activates NF-κB signaling, and increases the expression of NLRP3 and pro-IL-1β, thereby reinforcing inflammatory responses. In parallel, inflammasome-induced cellular stress exacerbates ROS production and mitochondrial damage, further promoting NLRP3 activation. Mitochondrial dysfunction, mtROS generation, and cytosolic release of mtDNA are now recognized as major upstream events in this process, with mtDNA acting as a DAMP that further sustains inflammasome signaling [21,22].

Amplification is also driven by pyroptotic cell death. Following inflammasome activation, caspase-1 processes pro-IL-1β into mature IL-1β, which is released through GSDMD-formed membrane pores during pyroptosis [23]. Pyroptotic cells simultaneously release DAMPs such as extracellular ATP and mtDNA, which activate P2X7 and PRRs, induce potassium efflux, and promote further NLRP3 activation [24,25]. Inflammasome activation additionally worsens mitochondrial dysfunction and ROS production, favoring NLRP3 oligomerization and inflammasome assembly [26].

TLR signaling is a major upstream regulator of NLRP3 priming and may be modulated by microRNAs under stress conditions, suggesting potential relevance for specific depression subtypes [27]. TLRs are expressed not only in peripheral immune cells but also in microglia, astrocytes, neurons, and oligodendrocytes. Through the adaptor myeloid differentiation primary response protein 88 (MyD88), TLR signaling promotes NF-κB activation, which induces NLRP3 transcription and enables inflammasome assembly [28]. Activated NLRP3 then promotes the maturation of pro-caspase-1 into active caspase-1, resulting in IL-1β and IL-18 processing and secretion and the propagation of inflammatory signaling [29].

Inflammasome activation has two major outcomes: caspase-1-dependent maturation of IL-1β and IL-18, and induction of pyroptosis, an inflammatory form of programmed cell death mediated by gasdermin pore formation. Pyroptosis can occur via inflammasome-dependent or independent pathways, but in the inflammasome-dependent setting it has emerged as a key driver of neuroinflammation and CNS pathology [30]. Pyroptosis in peripheral immune cells, endothelial cells, glial cells, and neurons has been implicated in the propagation of neuroinflammatory responses across multiple CNS disorders [31].

Mechanistically, NLRP3 oligomerizes with ASC and pro-caspase-1 to form the canonical inflammasome complex, enabling pro-caspase-1 autocatalytic maturation and subsequent cleavage of IL-1β and IL-18 precursors into their active forms [32,33]. Released IL-1β then signals through the IL-1 type I receptor complex, activating NF-κB and promoting the expression of additional inflammatory mediators, including IL-6, TNF-α, and prostaglandin E2 [34,35].

### 2.2. Models of NLRP3 Activation

**Calcium signaling model.** NLRP3 activation is closely linked to calcium signaling, and elevations in cytosolic Ca^2+^ promote inflammasome assembly by facilitating ASC recruitment to NLRP3 [19]. Endoplasmic reticulum Ca^2+^ release, regulated through phospholipase C-dependent signaling and inositol 1,4,5-trisphosphate receptors, further contributes to this process, while store-operated Ca^2+^ entry (SOCE) has also been implicated in NLRP3 activation [36]. In addition, disruption of Ca^2+^/cAMP homeostasis may favor inflammasome signaling. Mitochondrial calcium uptake links ER-derived Ca^2+^ flux to mitochondrial dysfunction, resulting in mitochondrial Ca^2+^ overload, increased mtROS production, loss of membrane potential, and subsequent NLRP3 activation [37,38].

**Reactive oxygen species model.** ROS are key signaling mediators in cellular homeostasis, but excessive ROS production promotes inflammasome activation. NADPH oxidase is a major source of cytosolic ROS, whereas mitochondria represent the principal intracellular source of ROS and a major contributor to oxidative stress in depression [39,40]. Inhibition of mitochondrial ROS suppresses NLRP3 activation and reduces the expression of NLRP3, ASC, caspase-1, and IL-1β [41,42].

Mechanistically, excessive ROS promotes dissociation of thioredoxin-interacting protein (TXNIP) from thioredoxin, enabling TXNIP to interact with the leucine-rich repeat domain of NLRP3 and trigger inflammasome activation [43]. Both mtROS and oxidized mtDNA released into the cytosol, as well as cardiolipin exposure, further contribute to NLRP3 activation [22,44].

**Potassium efflux model.** The ATP-gated purinergic receptor P2X7R acts as a cellular stress sensor and is widely expressed in immune and neural cell populations. During neuroinflammation, high extracellular ATP activates P2X7R and induces K^+^ efflux, a prerequisite for the interaction between NIMA-related kinase 7 (NEK7) and NLRP3 and thus for inflammasome assembly [45,46,47]. Beyond its established role in mitotic regulation, NEK7 has emerged as an essential mediator of NLRP3 activation. P2X7R signaling may also amplify inflammasome activity indirectly by promoting ROS generation and mitochondrial dysfunction [48].

**Chloride flux model.** Chloride signaling has also been implicated in inflammasome activation. P2X7R-dependent caspase-1 activation and IL-1β secretion may be facilitated by chloride flux, while the translocation of intracellular chloride channels such as CLIC1, CLIC4, and CLIC5 to the plasma membrane promotes NEK7–NLRP3 interaction and ASC oligomerization [20].

**Autophagy dysfunction.** Autophagy, the intracellular pathway responsible for degrading and recycling damaged cellular components, is altered in depression and may act as a negative regulator of NLRP3 inflammasome activity [49]. Because phosphorylated NLRP3 has been observed in association with autophagosomal compartments, autophagy may limit inflammasome signaling through degradation of inflammasome components [50]. Accordingly, inhibition of autophagy enhances NLRP3 activation [51].

## 3. Inflammasomes in Neuropsychiatric Disorders

The main mechanistic picture in neuropsychiatric disorders is no longer “monoamines only.” The stronger framework is a systems model in which stress biology, innate immune signaling, glial dysfunction, sleep–circadian disturbance, and impaired plasticity interact. Within that model, inflammasomes—especially NLRP3—and the orexin/hypocretin system are two important nodes rather than isolated pathways [52].

Several ongoing experimental and translational studies aim to clarify the mechanisms underlying neuropsychiatric disorders, with particular attention now being paid to systems that integrate arousal, stress, metabolism, and behavioral state. Among these, the orexin/hypocretin system has gained substantial acceptance as a major regulatory network rather than a sleep-specific pathway alone [53]. Orexin neurons, localized primarily in the lateral hypothalamus, project widely throughout the central nervous system and influence vigilance, emotional reactivity, reward-related behavior, autonomic responses, and cognitive flexibility. For this reason, orexinergic dysfunction has increasingly been implicated in a broad spectrum of neuropsychiatric and neurodegenerative conditions, including depression, anxiety disorders, addiction, schizophrenia (SZ)-related phenotypes, Alzheimer’s disease (AD), Parkinson’s disease (PD), and other disorders characterized by disturbed sleep–wake architecture and maladaptive stress responsivity [54].

Importantly, this orexin-centered view can be integrated with neuroinflammatory models of pathology [55]. Sleep loss, circadian disruption, and chronic stress—each tightly linked to orexin function—are now recognized to enhance central inflammatory signaling, in part through microglial activation, NF-κB-dependent priming, mitochondrial stress, and increased production of IL-1β and other pro-inflammatory cytokines [56]. These events converge on activation of the NLRP3 inflammasome, a key innate immune complex increasingly implicated in depression, anxiety-related behavior, cognitive decline, and neurodegenerative progression. Accordingly, the relationship between orexin dysfunction and neuropsychiatric disease may be interpreted not only in terms of altered arousal circuitry, but also in terms of its interaction with inflammasome-mediated neuroimmune dysregulation. In particular, a disturbed orexinergic state may amplify the consequences of fragmented sleep and chronic stress, thereby favoring NLRP3 activation, while persistent inflammasome signaling may in turn worsen vigilance instability, mood symptoms, and cognitive dysfunction. This bidirectional model provides a useful conceptual bridge between neuropeptide dysregulation and innate immune activation in complex brain disorders [57,58].

A comparative review is particularly useful because NLRP3 does not occupy the same pathogenic position in every disorder. In some conditions, especially Alzheimer’s disease, inflammasome activation is closely integrated with core lesion biology [59]. In others, such as mood disorders and PTSD (post-traumatic stress disorder), NLRP3 appears to function more as a stress-responsive amplifier of neuroimmune dysfunction. In schizophrenia and ASD (autism spectrum disorder), available evidence supports biologically plausible involvement, but direct causal centrality remains less firmly established [60,61].

### 3.1. Comparative Mechanistic Framework

Across the disorders addressed, a common logic can be described as follows: systemic or neural stress generates endogenous danger signals; glia undergo innate immune priming; NLRP3 assembles in response to intracellular disturbance; IL-1-family signaling and pyroptotic programs intensify inflammation; and downstream consequences manifest as synaptic loss, glial dysregulation, BBB (blood–brain barrier) or neurovascular perturbation, altered neurotransmission, and persistent behavioral phenotypes. What differs between diseases is the dominant entry point into this cascade and the main tissue-level consequence once inflammasome signaling is engaged.

### 3.2. Major Depressive Disorder (MDD)

In MDD, NLRP3 is best understood as a stress-to-cytokine transducer. Chronic psychosocial stress, glucocorticoid disequilibrium, excitatory overload, extracellular ATP, and oxidative stress converge on microglial priming and inflammasome assembly [60,62]. The ATP-P2X7 axis is especially prominent: stress-associated ATP release from neurons and astrocytes drives P2X7-dependent K^+^ efflux, NF-kB reinforcement, and IL-1b production, thereby linking cellular stress to depressive-like behavior in preclinical models [63,64].

Downstream consequences include reduced neurogenesis, impaired synaptic plasticity, perturbation of glutamatergic signaling, and inflammatory diversion of tryptophan metabolism into the kynurenine pathway. More recent work also implicates NLRP3-dependent pyroptosis as an escalation step that sustains stress-induced pathology [1,65]. The overall evidence base is strong in animal models and moderate in *human* translational studies, where inflammatory subgroups appear particularly relevant [66].

#### 3.2.1. *Human* Direct Evidence

The evidence for inflammasome involvement in MDD is stronger than in schizophrenia, but still less definitive than in AD. The most important direct *human* evidence comes from peripheral immune-cell studies. As summarized in a recent review, MDD patients showed enhanced expression of NLRP3 and caspase-1 in blood cells, with increased serum IL-1β and IL-18, and other studies reported significantly elevated NLRP3 and caspase-1 mRNA in MDD compared with healthy controls [60]. This matters because NLRP3 and caspase-1 are not generic inflammatory markers; they are core elements of the canonical inflammasome pathway.

Additional support comes from studies of plasma and PBMC inflammatory components discussed in the postmortem/suicide literature. Pandey et al. [67] note prior reports of higher NLRP3 and ASC mRNA in PBMCs (peripheral blood mononuclear cells) and increased IL-1β and IL-18 protein in serum of MDD patients, as well as increased ASC, caspase-1, and IL-18 in plasma, though IL-1β was not always consistently elevated across studies. This pattern supports the idea that at least a subset of patients with depression exhibits a circulating inflammasome-activated phenotype.

Direct central evidence also exists, although it is still limited and complicated by the frequent use of suicide postmortem samples rather than broad MDD cohorts. In postmortem prefrontal cortex from depressed individuals who died by suicide, ASC, NLRP3, NLRP1, NLRP6, and caspase-3 mRNA and protein were increased, together with elevated IL-1β, TNF-α, and IL-6 [60]. Pandey et al. [67] further reported upregulated NLRPs and hyperactive inflammasomes in postmortem brains of depressed suicide cases, strengthening the argument that inflammasome-related signaling is present centrally and not only in peripheral blood [68]. However, because these samples combine depression with suicide, they do not prove that NLRP3 activation is universal across all MDD presentations.

Overall, the direct evidence in MDD is meaningful: the field has moved beyond simple cytokine correlations into demonstrable changes in NLRP3, ASC, caspase-1, IL-1β, and IL-18 in patient-derived material. Still, the strongest direct evidence remains peripheral and postmortem rather than in vivo.

#### 3.2.2. *Human* Indirect Evidence

The circumstantial case in MDD is extensive. Depression is one of the psychiatric disorders most consistently linked to elevated inflammatory mediators such as C-reactive protein (CRP), IL-6, IL-1β, and TNF-α, together with microglial activation, HPA-axis dysregulation, kynurenine pathway activation, oxidative stress, and altered brain network structure [60,69]. These findings are compatible with NLRP3 activation because NLRP3 sits upstream of mature IL-1β/IL-18 release and is highly responsive to stress-related mitochondrial dysfunction, ROS, DAMPs, and glucocorticoid-associated immune priming.

A particularly interesting indirect line of evidence involves NLRP3 gene methylation and cortical thickness. Han et al. [70] reported that methylation changes in the NLRP3 gene were associated with cortical thickness abnormalities in major depressive disorder (MDD), suggesting that inflammasome-related biology may intersect with structural brain alterations rather than acting only as a peripheral immune marker. This does not directly prove inflammasome activation, but it supports the idea that NLRP3-related pathways may be biologically embedded in disease-relevant brain phenotypes.

The broader MDD literature also consistently links chronic stress to microglial activation in the hippocampus, medial prefrontal cortex, anterior cingulate, and amygdala, all of which are regions strongly implicated in depression [60,71,72]. Because stress is a major upstream trigger of mitochondrial dysfunction, ROS production, and NF-κB priming, it provides a plausible mechanistic bridge between environmental vulnerability and NLRP3 activation.

Thus, the indirect evidence in MDD is strong, but it remains indirect whenever it relies on inflammatory cytokines, brain imaging, methylation, or stress biology without directly measuring inflammasome assembly or function.

#### 3.2.3. Animal Causal Evidence

The causal animal evidence for MDD is considerably more developed than in schizophrenia and is one of the strongest reasons the NLRP3 hypothesis has gained traction in depression research [73]. In a chronic stress context, beta-hydroxybutyrate (BHB)—described as an endogenous NLRP3 inflammasome inhibitor—attenuated stress-induced behavioral and inflammatory responses, and the authors explicitly placed NLRP3 and caspase-1 upstream of IL-1β maturation relevant to depressive-like behavior [74]. Their study also emphasized prior evidence that IL-1β neutralization blocks depressive-like behaviors induced by chronic unpredictable stress, reinforcing the logic that the inflammasome–IL-1β axis is causally relevant.

A second key causal study showed that NLRP3 contributes to LPS-induced depressive-like behaviors via indoleamine 2,3-dioxygenase (IDO) induction [75]. This is important because it links inflammasome biology not only to cytokine production but also to a classic depression-relevant metabolic pathway: tryptophan catabolism through IDO and the kynurenine system.

The preclinical literature summarized in more recent reviews is even broader. CUMS and related chronic stress models consistently show increased NLRP3, ASC, caspase-1, IL-1β, and IL-18 in depression-relevant brain regions, and depressive-like behaviors are ameliorated when NLRP3 signaling is inhibited pharmacologically or genetically [65,76]. Wan et al. [65] specifically note that knockdown of *GSDMD*, *caspase-1*, and astrocyte *NLRP3* genes attenuated depression-like manifestations, bringing pyroptosis into the mechanistic picture.

Taken together, the animal data show what is still missing in many *human* studies: loss-of-function or pharmacologic suppression of the inflammasome pathway improves depressive-like phenotypes. For that reason, the causal preclinical evidence for NLRP3 in MDD is substantially stronger than the *human* interventional evidence.

#### 3.2.4. Major Gaps

Despite the strong mechanistic and preclinical literature, several major gaps remain. First, depression is highly heterogeneous, and not all patients show an inflammatory phenotype. This raises the possibility that NLRP3 is relevant primarily to an immune–inflammatory subtype of MDD rather than to the disorder as a whole. Second, many *human* studies still rely on peripheral blood and serum markers, leaving open the question of how well these signals reflect brain inflammasome activity. Third, the postmortem literature is limited and confounded by suicide, medication exposure, medical comorbidity, obesity, smoking, and cause of death. Fourth, although NLRP3-related markers are increased in MDD, it remains difficult to determine whether inflammasome activation is a primary driver, a downstream consequence of stress and metabolic dysfunction, or both. Finally, there are still no definitive clinical trials in MDD directly testing selective NLRP3 inhibitors as antidepressant agents.

### 3.3. Bipolar Disorder (BD)

In BD, inflammasome biology is tightly intertwined with mitochondrial and metabolic pathology. Mitochondria-associated membranes provide a subcellular platform for NLRP3 assembly, allowing ROS excess, altered Ca^2+^ handling, electron transport chain inefficiency, and bioenergetic instability to feed directly into inflammatory signaling [77,78]. This architecture distinguishes BD from MDD, where psychosocial stress and purinergic signaling are more explicitly foregrounded.

The strongest conceptual model in BD is that NLRP3 operates as an immunometabolic sensor that links mitochondrial injury to episode sensitization, white matter dysfunction, altered connectivity, and cognitive burden. Evidence suggests that NLRP3-related biology may be particularly relevant in inflammatory-metabolic subgroups and in individuals with recent episodes, psychosis, obesity, or insulin resistance [77,78,79]. Although direct *human* mechanistic proof remains less developed than in AD or MDD, the biological rationale is coherent and increasingly supported.

#### 3.3.1. *Human* Direct Evidence

Despite a clear line of direct peripheral evidence, in vivo brain validation for inflammasome involvement in bipolar disorder (BD) remains limited. In a PBMC-based analysis, Scaini et al. [80], reported upregulation of TSPO-related proteins, downregulation of mitophagy-related proteins, and activation of the NLRP3 inflammasome in patients with bipolar disorder type I. NLRP3-related changes were not described as isolated inflammatory abnormalities, but as part of a broader mitochondrial stress signature involving altered 18 kDa Translocator Protein (TSPO)/Voltage-Dependent Anion Channel 1 (VDAC1) signaling, impaired mitophagic quality control, and correlations with manic and depressive symptom severity. NLRP3-related molecular changes are measured in patient-derived cells, representing a translational tool to clinical state.

The direct evidence base has recently broadened further through *human* stem-cell disease models. A translational psychiatry study [81] using iPSC-derived cerebral organoids reported mitochondrial and inflammatory vulnerability in bipolar disorder and explicitly referenced prior evidence for increased NLRP3 inflammasome activation in prefrontal postmortem samples and PBMCs from BD patients. Thus, inflammasome-associated stress biology is not confined to peripheral immune cells and may map onto disease-relevant neural systems, but direct evidence remains incomplete. Most *human* BD studies do not yet assess the full canonical inflammasome sequence (NLRP3 assembly, ASC speck formation, caspase-1 cleavage, mature IL-1β/IL-18 release, and pyroptotic signaling) in the same sample. Thus, while BD now has credible direct evidence for NLRP3-related activation in peripheral cells, it still lacks the level of convergent multi-compartment validation that would allow inflammasome activation to be considered a settled core mechanism.

#### 3.3.2. *Human* Indirect Evidence

A large literature has documented elevated peripheral inflammatory markers across mood states, including IL-1β, IL-6, TNF-α, soluble cytokine receptors, and other innate immune mediators, particularly during acute mania and depression [77,82]. These findings are important because IL-1β and IL-18 are classical downstream products of inflammasome activation, but most studies do not directly establish that their increase is specifically NLRP3-dependent rather than the product of broader immune activation.

The link becomes more persuasive when inflammatory findings are integrated with mitochondrial dysfunction. Bipolar disorder is one of the psychiatric conditions in which evidence for altered energy metabolism, oxidative stress, lactate accumulation, calcium dysregulation, and mitochondrial structural abnormalities is particularly strong [78,83]. This matters because NLRP3 is a danger-sensing inflammasome strongly responsive to ROS, mitochondrial dysfunction, oxidized mtDNA, ATP signaling, and defective mitophagy. For this reason, the mitochondrial and inflammatory hypotheses of BD are not parallel studies but increasingly overlapping ones [83].

Postmortem evidence also supports a neuroimmune contribution, but without full inflammasome specificity. Reviews of BD brain tissue studies conclude that many postmortem investigations show evidence of neuroinflammation and microglial abnormalities, but findings vary by region, disease phase, treatment history, comorbidity, and tissue quality [78,84]. The literature includes reports of changes in IL-1 pathway markers and microglial activation in prefrontal cortex, but there is still relatively little direct measurement of canonical NLRP3 complex activation in *human* BD brain [78,83]. A further indirect line of support comes from pharmacology. Several agents with anti-inflammatory or inflammasome-interactive properties—such as allopurinol, pioglitazone, and statins—have shown efficacy signals in selected BD subpopulations or symptom domains, particularly acute mania, bipolar depression, or cognition [77]. These clinical findings do not prove NLRP3 causality, but they are consistent with the idea that inflammatory and metabolic stress pathways contribute to illness expression in at least a subgroup of patients.

#### 3.3.3. Animal Causal Evidence

The animal literature in BD is mixed and is notable for both positive and negative findings. On the positive side, preclinical theories have long proposed that mitochondrial dysfunction and oxidative stress in BD could converge on inflammasome activation, especially NLRP3, via enhanced ROS production and impaired cellular quality control [83]. This framework fits well with the broader mood disorder literature and with the observation that mood stabilizers such as lithium have immunomodulatory and mitochondrial effects that could, in principle, restrain inflammasome-associated signaling [78].

However, causal animal evidence remains less mature than might be expected from the strength of the mechanistic model. A particularly important negative result was reported by Farooqui et al., who found no NLRP3 inflammasome expression in the ouabain animal model of bipolar disorder [85]. This study is valuable because it prevents overstatement: not all accepted BD-related animal models recapitulate NLRP3 activation, and inflammatory changes in BD cannot simply be assumed to be universal or primary. The failure to detect NLRP3 in the ouabain model argues against a simplistic view in which inflammasome activation is automatically present in every mania-like state.

Thus, the current animal evidence is best interpreted as supportive but not decisive. Some models and mechanistic frameworks are highly compatible with NLRP3 activation, especially those emphasizing mitochondrial dysfunction and oxidative injury, yet at least one established BD model does not show NLRP3 expression [85]. This heterogeneity strongly suggests that inflammasome involvement in BD may be state-dependent, model-dependent, or subgroup-dependent rather than universal.

#### 3.3.4. Major Gaps

Several key gaps remain. First, BD lacks robust in vivo demonstration of NLRP3 activation in the brain across illness phases. Peripheral evidence is stronger than central evidence, but PBMC findings alone cannot establish whether inflammasome dysregulation is causal, compensatory, or epiphenomenal. Second, many studies infer inflammasome involvement from cytokines or mitochondrial stress without directly measuring ASC specks, cleaved caspase-1, mature IL-1β/IL-18, or pyroptosis markers. Third, the heterogeneity of bipolar disorder likely obscures signal; manic, depressive, mixed, and euthymic states may differ substantially in inflammatory biology, and BD-I may not behave the same as BD-II. Fourth, current animal models are inconsistent, as illustrated by the negative ouabain study, and therefore do not yet provide a stable causal platform [85]. Finally, clinical confounders—including obesity, smoking, metabolic syndrome, sleep disruption, medication exposure, and medical comorbidity—are particularly important in BD because each can independently activate inflammatory and NLRP3-related pathways [77,78].

### 3.4. Schizophrenia (SZ)

For SZ, inflammasome activation is most plausibly embedded within a broader framework of aberrant microglia–neuron interaction, complement-linked synaptic elimination, and neuronal stress signaling. iPSC (induced pluripotent stem cells)-based studies indicate that patient-derived microglia can display enhanced activation, elevated TNFα and NF-kB signaling, and upregulation of inflammasome-related genes, including *NLRP2* and *NLRP3*, whereas patient-derived neurons show reduced presynaptic integrity [86,87].

Thus, NLRP3 in SZ is better regarded as a contributor to maladaptive microglial state transitions than as a standalone disease engine. Mitochondrial dysfunction, ROS, HMGB1, and other neuronal DAMPs may provide upstream priming and activation signals, while complement biology biases the system toward excessive or mistimed synaptic pruning [86,87,88]. The evidence is biologically persuasive but still less direct and less mature than in AD or mood disorders.

#### 3.4.1. *Human* Direct Evidence

Direct evidence linking inflammasome signaling to SZ remains limited. The strongest SZ-specific data come from patient-derived cellular systems rather than from in vivo brain measurements. In an induced pluripotent stem cell model, SZ-derived microglia-like cells displayed an enhanced inflammatory phenotype with increased TNFα secretion, elevated NF-κB signaling, upregulation of *NLRP2* and *NLRP3*, and, importantly, increased caspase-1 activity relative to control cells; these changes co-occurred with impaired neuron–microglia interactions and reduced presynaptic density, directly connecting inflammasome-related activity to synaptic pathology relevant to SZ [86]. This study currently provides the clearest disease-relevant direct evidence because it moves beyond generic cytokine elevation to a functional inflammasome readout.

A second line of direct mechanistic evidence comes from microglial inflammatory models designed around SZ-relevant immune activation. In primary microglia and BV-2 cells exposed to poly(I:C), clozapine reduced expression of NLRP3, ASC, and pro-caspase-1, while also reducing caspase-1 activity; the experimental framework explicitly compared these effects with CRID3-mediated NLRP3 inhibition logic, supporting the idea that at least part of the anti-inflammatory effect of clozapine converges on the NLRP3 pathway [89]. This is direct evidence for pathway engagement, although it is still model-based rather than patient in vivo proof.

Less direct, but still important, are older *human* studies showing increased IL-1β production in SZ. A genetic/immunologic study summarized prior reports of elevated CSF IL-1β in first-episode SZ, increased monocyte IL-1β release before treatment, and PBMC IL-1β overexpression in patients and even siblings [90]. These findings are highly compatible with inflammasome activation, but because they did not directly establish NLRP3 assembly, ASC speck formation, or caspase-1-dependent cytokine maturation, they should be interpreted as partially direct rather than definitive inflammasome proof.

#### 3.4.2. *Human* Indirect Evidence

Peripheral immune activation is one of the most replicated biological findings in SZ, especially in first-episode psychosis and acute relapse. Elevated inflammatory mediators including IL-1β, IL-6, TNF-α, MCP-1 (monocyte chemoattractant protein-1), and YKL-40 (chitinase-3-like protein 1), together with increased monocyte numbers, support the presence of an activated innate immune state, but these changes are not specific to inflammasomes and may arise from multiple inflammatory programs [91,92].

Postmortem studies also support cerebral immune dysregulation, but again with limited inflammasome specificity. A systematic review of postmortem SZ brains found evidence for inflammatory alterations in subsets of patients, yet the findings were heterogeneous across markers and regions [92]. IL-1β changes were inconsistent: some studies reported frontal cortical increases, whereas others found no significant change, making it difficult to conclude that inflammasome activation is a universal feature of schizophrenia brain tissue.

Additional circumstantial support comes from purinergic biology. Altered purinergic receptor expression has been identified in SZ frontal cortex, including changes in P2RX4 and P2RX7-related signaling context; this is mechanistically relevant because extracellular ATP and purinergic receptor activation are canonical upstream triggers of microglial NLRP3 activation [93]. Likewise, mitochondrial dysfunction, oxidative stress, and release of damage-associated molecular patterns are repeatedly discussed as plausible inflammasome triggers in SZ models and patient-derived cells, but they remain upstream stress signals rather than direct evidence of NLRP3 activation itself [86].

#### 3.4.3. Animal Causal Evidence

Compared with AD, stroke, or major depression, causal animal evidence for NLRP3 in SZ is still relatively underdeveloped. The main value of animal work lies in showing that SZ-relevant inflammatory contexts can engage inflammasome-like biology and that intervention can modify those responses. The strongest such evidence comes from poly(I:C)-based microglial models, where clozapine attenuated NLRP3-pathway activation markers and caspase-1 activity [89]. Because poly(I:C) is widely used to model infection-related neurodevelopmental risk and maternal immune activation, these findings fit well with neurodevelopmental hypotheses of SZ.

Broader SZ-relevant animal frameworks, such as maternal immune activation, MK-801, PCP, and ketamine models, clearly demonstrate microglial activation, oxidative stress, and neurodevelopmental immune abnormalities, but most do not directly measure the canonical NLRP3 axis. Reviews of maternal immune activation emphasize robust microglial and inflammatory abnormalities in offspring and strong construct validity for SZ-related phenotypes, yet they generally stop short of showing NLRP3-specific rescue experiments [94,95]. Thus, these models provide strong causal evidence for immune involvement in SZ-like phenotypes, but only circumstantial evidence for NLRP3 unless inflammasome-specific readouts are included.

Overall, the animal literature supports a model in which SZ-relevant insults create a permissive environment for inflammasome activation through mitochondrial stress, ROS production, DAMP release, and microglial priming, but it has not yet delivered a large body of SZ-specific studies in which *NLRP3* knockout or selective NLRP3 inhibition robustly rescues core behavioral and synaptic phenotypes.

#### 3.4.4. Major Gaps

First, there is no definitive in vivo demonstration that NLRP3 activation is a core and reproducible feature of SZ brain pathology across patient subgroups. Second, postmortem evidence remains heterogeneous and is confounded by medication exposure, smoking, metabolic disease, substance use, agonal state, and tissue quality. Third, peripheral cytokine findings, though compelling, are not specific enough to distinguish inflammasome signaling from broader innate immune activation. Fourth, SZ remains biologically heterogeneous, and it is likely that inflammasome activation characterizes only a subset of patients with an inflammatory biotype rather than the entire disorder. Finally, there are still no established clinical trials in SZ specifically targeting NLRP3, ASC, caspase-1, or the downstream IL-1/IL-18 axis.

### 3.5. Post-Traumatic Stress Disorder (PTSD)

PTSD extends the stress–inflammasome concept into the domain of trauma-related circuit pathology. Current models indicate that severe or prolonged trauma promotes glutamatergic dysregulation, astrocytic ATP release, microglial P2X7 activation, oxidative stress, HMGB1-TLR4-RAGE signaling, and, in susceptible settings, persistent NLRP3 engagement in stress-sensitive regions such as the amygdala, hippocampus, and prefrontal cortex [96,97,98]. PTSD also appears unusually sensitive to HPA-axis abnormalities and mitochondrial dysfunction, both of which can lower the threshold for inflammasome activation [1,97].

Compared with MDD, PTSD places greater emphasis on fear-memory circuitry, extinction deficits, hyperarousal, and trauma-triggered noradrenergic dysregulation. Nonetheless, the shared architecture is substantial: ATP-P2X7 signaling, ROS generation, IL-1-family cytokine maturation, and glia-neuron crosstalk recur across both disorders [1,96,99]. Evidence for PTSD is moderate overall, with a stronger preclinical than clinical basis. The literature supports NLRP3 as a meaningful pathogenic amplifier rather than a fully validated universal driver [100].

**Inflammasome involvement in post-traumatic stress disorder: emphasis on the NLRP3 axis**.

#### 3.5.1. *Human* Direct Evidence

Compared with AD and even major depressive disorder, the evidence for direct NLRP3 inflammasome involvement in post-traumatic stress disorder is still relatively limited. The strongest *human* literature in PTSD has focused more on inflammatory cytokines than on direct measurement of inflammasome components such as NLRP3, ASC, cleaved caspase-1, or mature IL-18. A systematic review of IL-1β in PTSD found that roughly half of the cross-sectional *human* studies reported elevated plasma or serum IL-1β in PTSD, and some studies also found increased spontaneous IL-1β production, indicating that trauma-related inflammatory activation is present in at least a subset of patients [101]. However, this does not by itself prove NLRP3 activation, because IL-1β can be increased through multiple inflammatory pathways.

A broader review of neuroinflammation in PTSD [102] similarly concluded that combat-related PTSD cohorts show elevated pro-inflammatory composites including IL-1β, IL-6, TNF-α, IFN-γ, and CRP, and that these elevations persist even after adjustment for major confounders such as early-life trauma, BMI, medications, and comorbid depression severity in some studies. These findings provide strong support for immune activation in PTSD, but they remain mostly circumstantial with respect to inflammasome biology.

At present, there is no strong *human* PTSD study equivalent to the PBMC NLRP3/caspase-1 papers seen in MDD or BD. For PTSD, the *human* data support IL-1β-rich inflammation, but do not yet robustly demonstrate a full canonical NLRP3 → ASC → caspase-1 → IL-1β/IL-18 sequence in patient-derived cells or tissue [101]. This distinction is important: PTSD has credible inflammatory evidence, but its direct inflammasome evidence is still thinner than for MDD, BD, or AD.

#### 3.5.2. *Human* Indirect Evidence

The circumstantial case for inflammasome involvement in PTSD is much stronger. PTSD is consistently linked to chronic low-grade inflammation, altered innate and adaptive immune signaling, HPA-axis dysregulation, sympathetic overactivation, sleep disruption, and mitochondrial stress. Because all of these processes can prime or activate the NLRP3 inflammasome, they create a strong biological context in which inflammasome activation is plausible.

Lee et al. [102] emphasize that PTSD is associated with elevated inflammatory mediators such as CRP, IL-1β, IL-6, and TNF-α, especially in combat-exposed cohorts, and that this inflammatory phenotype may be a risk factor rather than merely a downstream consequence of trauma exposure. Similarly, Hori and Kim [103] summarizes a wide body of evidence linking PTSD to persistent immune dysregulation and elevated inflammatory markers across both civilian and military populations.

A more mechanistic indirect line comes from the emerging mitochondrial literature. Dmytriv et al. [97] explicitly frame mitochondrial dysfunction as a possible trigger of neuroinflammation in PTSD, which is highly relevant because mitochondrial ROS, ATP release, oxidized mtDNA, and related DAMPs are classic upstream activators of NLRP3. This does not prove inflammasome activation in PTSD patients, but it provides a credible mechanistic bridge between traumatic stress, cellular danger signaling, and innate immune activation.

Indirect evidence in PTSD shows that the disorder is very convincingly inflammatory, and several of the implicated upstream drivers are well known to converge on NLRP3 biology; the *human* literature is still more focused on measured cytokines and stress physiology than direct inflammasome machinery.

#### 3.5.3. Animal Causal Evidence

The animal evidence for PTSD is more informative with respect to NLRP3 than the *human* data. Yamanashi et al. [104] used a *rodent* single prolonged stress (SPS) PTSD model and tested beta-hydroxybutyrate (BHB), which they describe as an endogenous NLRP3 inflammasome inhibitor. They found that BHB attenuated PTSD-like anxiety-related behavior, providing causal support for the idea that NLRP3-linked inflammatory signaling contributes to trauma-related behavioral abnormalities. Interestingly, in that study serum IL-1β remained elevated after SPS and was not significantly normalized by BHB, which suggests that behavioral rescue may occur even without a complete normalization of peripheral cytokines, or that central and peripheral inflammatory changes may dissociate [104].

This PTSD animal evidence is important because it moves beyond correlation: it shows that modulating an endogenous NLRP3 inhibitor can alter trauma-related phenotype expression. However, it is still not equivalent to a clean *NLRP3* knockout rescue experiment in a PTSD model. So the causal evidence is meaningful, but not yet as strong as the *APP/PS1* knockout literature in AD [105,106] or the broader genetic/pharmacologic *NLRP3* suppression literature in depression [107,108].

More broadly, PTSD animal models are repeatedly described as inflammatory and immune-reactive, with stress-induced alterations in fear memory, neuroendocrine function, and glial biology [102]. Many of these models are still interpreted through general inflammation rather than explicit inflammasome readouts. The field therefore has promising causal signals, but not yet a large body of PTSD-specific experiments in which *NLRP3* deletion, *ASC* deletion, or caspase-1 inhibition reproducibly rescues the phenotype [109].

#### 3.5.4. Major Gaps

Direct *human* evidence is limited: PTSD lacks strong PBMC, monocyte, or postmortem studies showing reproducible increases in NLRP3, ASC, cleaved caspase-1, or mature IL-18 analogous to those available in MDD or AD. The current *human* literature often cannot distinguish whether elevated IL-1β reflects true inflammasome activation or broader immune stress. PTSD is itself heterogeneous, spanning combat trauma, sexual violence, disaster exposure, childhood trauma, and TBI-associated forms, which likely differ biologically. Also, peripheral immune studies are heavily confounded by sleep disturbance, metabolic disease, smoking, substance use, chronic pain, and comorbid depression. Finally, there are no established clinical trials in PTSD directly testing selective NLRP3 inhibitors or related inflammasome-targeted drugs.

### 3.6. Alzheimer’s Disease (AD)

AD is the condition in which NLRP3 occupies the most central and causally mature mechanistic position. Proteinopathy itself acts as a disease-specific danger signal. Amyloid-b uptake, fibrillar Aβ, tau species, lysosomal stress, mitochondrial dysfunction, and impaired autophagic flux activate microglial NLRP3, resulting in caspase-1 activation, IL-1b/IL-18 maturation, and pyroptotic amplification [110,111,112].

**Figure 2:**
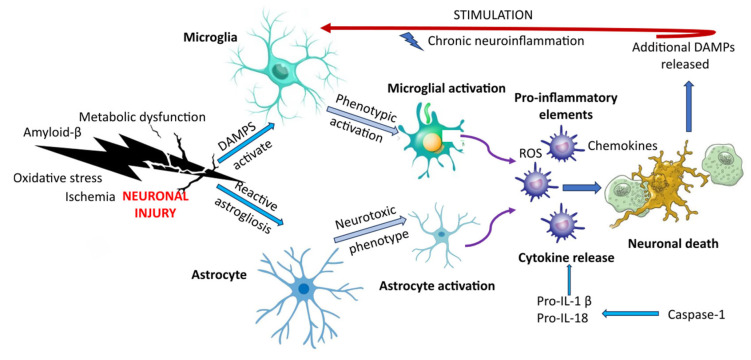
Neuroinflammatory cascade following neuronal injury. DAMPs—damage-associated molecular patterns; ROS—reactive oxygen species; Pro-IL-1β—pro interleukin 1β; Pro-IL-18—pro interleukin 18.

Neuronal damage, neuroinflammation, and neuronal death are all connected by a series of cellular and molecular processes (Figure 2). Microglia and astrocytes mediate the immune response of the central nervous system in AD, which is known as neuroinflammation.

Damage-associated molecular patterns (DAMPs) are released when neuronal damage is caused by pathological stimuli such as amyloid-β accumulation, metabolic dysfunction, oxidative stress, and ischemia. Glial activation results from pattern recognition receptors on microglia and astrocytes recognizing these endogenous danger signals. Reactive oxygen species (ROS), chemokines, and inflammatory mediators are produced when microglia undergo phenotypic activation and take on a pro-inflammatory state.

Concurrently, astrocytes may develop a neurotoxic phenotype that exacerbates the inflammatory conditions in response to injury signals through reactive astrogliosis. Pro-IL-1β and pro-IL-18 are cleaved by caspase-1 upon inflammasome activation, which increases the release of cytokines and amplifies inflammatory signaling. Inflammatory mediators derived from microglia and astrocytes work together to cause neuronal dysfunction and, eventually, neuronal death. A feed-forward cycle of glial activation and progressive neurodegeneration is reinforced by the release of additional DAMPs by degenerating neurons, which maintains chronic neuroinflammation.

A major AD-specific feature is the release of extracellular ASC specks, which bind Aβ and promote further amyloid aggregation, thereby converting inflammasome activation into a direct feed-forward driver of lesion propagation. NLRP3 also exhibits a bidirectional relationship with tau pathology [113]: tau can activate inflammasome signaling, while inflammasome activity can exacerbate tau phosphorylation and accumulation [114,115]. The result is impaired Aβ clearance, maladaptive microglial polarization, synapse loss, and progressive cognitive decline. Among all disorders reviewed here, AD has the strongest evidence for direct therapeutic targeting of NLRP3 [116,117].

#### 3.6.1. *Human* Direct Evidence

Among neuropsychiatric and neurodegenerative disorders, AD provides one of the strongest *human* datasets implicating inflammasome biology, although most direct evidence still comes from peripheral immune cells and postmortem tissue rather than real-time in vivo brain assays. A particularly important study by Saresella et al. [118] showed that NLRP3 and NLRP1 inflammasomes are activated in AD, with increased expression of inflammasome-related transcripts and proteins in patient monocytes, together with enhanced co-localization of NLRP3/PYCARD and NLRP1/caspase-1 after LPS priming and Aβ42 stimulation [118]. This goes beyond generic cytokine elevation and demonstrates disease-associated activation of canonical inflammasome machinery in cells derived from AD patients.

Clinical study evidence is also substantial, although less abundant than the mouse literature. Heneka et al. explicitly reported that cleaved caspase-1 was increased in AD patients, a finding highlighted in subsequent commentary as evidence of disease-associated inflammasome activation in the *human* brain [119]. Because cleaved caspase-1 is a functional hallmark of inflammasome activation, this places AD in a stronger position than SZ or BD, where direct brain-level inflammasome readouts are still sparse. Taken together, the available *human* data support the conclusion that NLRP3 activation in AD is not merely theoretical or inferred from cytokines, but is demonstrable in patient-derived cells and in *human* disease tissue.

#### 3.6.2. *Human* Indirect Evidence

The indirect evidence in AD is extensive and convergent. Neuroinflammation has long been recognized as a major component of AD, and NLRP3 fits naturally into this framework because both amyloid-β (Aβ) and tau-associated stress can activate microglial inflammatory programs. Reviews of AD-NLRP3 signaling consistently describe the pathway as biologically integrated with plaque-associated microglial activation, IL-1β/IL-18 maturation, mitochondrial dysfunction, NF-κB priming, and progressive synaptic and neuronal injury [114,120,121].

Aβ provides one of the strongest mechanistic circumstantial links. As summarized in review and commentary literature, aggregated Aβ functions as an endogenous danger signal capable of activating NLRP3 in microglia [114,122]. Likewise, tau pathology is now increasingly tied to NLRP3 signaling. In the influential study by Ising et al. [113], the authors note that prior work had already shown elevated active caspase-1 in plaque-containing mice and AD patients, and then demonstrate that tau monomers and oligomers can stimulate IL-1β secretion in an ASC- and NLRP3-dependent manner. These observations strongly support the idea that NLRP3 is not only linked to amyloid pathology, but may also sit at the interface between amyloid-driven inflammation and downstream tau propagation.

Thus, the indirect evidence in AD is unusually strong because the main pathological hallmarks of the disease—Aβ, tau, microglial activation, mitochondrial stress, and cytokine maturation—map coherently onto known NLRP3 biology. Still, these lines of evidence remain indirect whenever they do not directly assess inflammasome assembly or function in the same experimental system.

#### 3.6.3. Animal Causal Evidence

The strongest case for NLRP3 in AD comes from animal studies. Heneka et al. [123] demonstrated that NLRP3 is activated in AD and contributes to pathology in APP/PS1 mice. In that study, APP/PS1/NLRP3^−/−^ and APP/PS1/Caspase-1^−/−^ *mice* showed markedly reduced hippocampal and cortical Aβ deposition, and the authors also reported improved cognitive performance together with increased levels of the Aβ-degrading enzyme IDE. In the context of evidence grading, this is one of the clearest demonstrations in the neuroinflammation field that loss of NLRP3 signaling modifies core disease pathology.

Ising et al. [113] showed that NLRP3 inflammasome activation drives tau pathology. Their work demonstrated that tau monomers and oligomers induce NLRP3-dependent IL-1β secretion and that inhibition of NLRP3 signaling reduces tau phosphorylation and aggregation. Importantly, the study linked Aβ-driven NLRP3 activation to downstream tau pathology, thereby placing inflammasome biology in a central mechanistic position rather than at the edge of the disease cascade. This is highly significant because it suggests NLRP3 participates not only in inflammatory amplification, but in the pathological transition from amyloid accumulation to tau-mediated neurodegeneration.

For AD, therefore, the animal literature supplies what is still missing in most psychiatric disorders: reproducible evidence that genetic suppression of *NLRP3* or *caspase-1* improves pathology and behavior in disease-relevant *mouse* models.

#### 3.6.4. Major Gaps

There is still no definitive clinical demonstration that NLRP3 inhibition slows cognitive decline in *human* AD. Although *human* monocyte and postmortem findings are compelling, routine in vivo biomarkers of NLRP3 activation in living AD patients are still lacking. It is not clear how early inflammasome activation occurs relative to amyloid deposition, tau spread, and clinical conversion from mild cognitive impairment to dementia. There is a need for better clarification of cellular specificity—especially the relative contributions of microglia, infiltrating myeloid cells, astrocytes, and possibly neurons—to inflammasome signaling at different disease stages.

Another unresolved issue is translational heterogeneity. The *mouse* data are exceptionally strong, but many anti-inflammatory strategies in AD have failed historically when moved into the clinic. Therefore, even though AD currently offers some of the best support for NLRP3 involvement in any brain disorder, the pathway still awaits definitive clinical validation.

### 3.7. Autism Spectrum Disorder (ASD)

ASD presents the most heterogeneous and developmentally contingent case. Neuroinflammation and glial dysregulation are repeatedly reported, but NLRP3 is not yet established as a universal or primary disease engine. Instead, developmental immune misprogramming appears more central: maternal immune activation, prenatal stress, gut-immune perturbation, altered cytokine tone, and genetic susceptibility shape microglial maturation and synaptic pruning trajectories [61,124,125].

Inflammasome-related mechanisms likely contribute to neuroimmune ASD subgroups rather than the entire spectrum. In ASD, the pathogenic question is not merely whether NLRP3 is active in the mature brain, but whether early-life immune signaling alters microglial developmental roles in a durable way, leading to E/I imbalance, pruning deficits, and abnormal circuit formation [61,126]. Direct mechanistic evidence for NLRP3 centrality remains weaker than for AD, MDD, PTSD, or BD.

#### 3.7.1. *Human* Direct Evidence

The evidence for inflammasome involvement in ASD is suggestive and increasingly interesting, but overall, it is less mature than in AD and less systematically documented than in MDD. The strongest direct *human* evidence lies more on the downstream IL-18/IL-18R branch than on a full canonical NLRP3–ASC–caspase-1 demonstration.

A key postmortem study by Tsilioni et al. [125] showed that the amygdala and dorsolateral prefrontal cortex of children with ASD had increased expression of IL-18 and IL-18R, alongside increased IL-37, an anti-inflammatory IL-1 family cytokine that functionally interfaces with the IL-18 system. This is important because IL-18 is a classic inflammasome-processed cytokine, and its elevation in brain regions is closely linked to social behavior and higher cognition places inflammasome-relevant signaling directly within ASD brain tissue rather than only in peripheral blood. However, this study did not directly show NLRP3 assembly, ASC speck formation, or cleaved caspase-1 in the same samples.

There is also evidence of a much more direct peripheral inflammasome phenotype in ASD. A 2016 study [127] found markedly elevated *NLRP3*, *caspase-1*, *IL-1β*, and *IL-18* gene expression in baseline and LPS/ATP-stimulated monocytes from ASD subjects, together with increased serum IL-1β and IL-18 and elevated caspase-1 activity.

Therefore, ASD has direct evidence for inflammasome-relevant cytokine dysregulation in the brain and likely has direct peripheral NLRP3-pathway activation evidence, but the latter remains less securely documented in this review.

#### 3.7.2. *Human* Indirect Evidence

ASD has one of the more substantial neuroinflammation studiy documentation [128], with repeated reports of altered cytokine profiles in serum, CSF, brain tissue, and immune cells, including IL-1β, TNF, CXCL8, IL-6, IL-17, and IL-18. Tsilioni et al. [125] explicitly note that inflammatory molecules such as IL-1β are increased in the serum, CSF, and brain of many ASD patients. Similarly, recent cytokine work in children with ASD continues to support an altered plasma inflammatory profile [129].

Postmortem and neuroimaging work also point toward chronic glial activation. Older autism neuropathology reports, as summarized in broader reviews, describe increased microglial density and activation in multiple brain regions, including cerebellar and cortical areas, while PET work has also supported excessive microglial activation in some ASD cohorts [130,131]. These data do not establish inflammasomes specifically, but they create a strong context for inflammasome plausibility, because microglia are the principal CNS cell type in which NLRP3 is typically studied.

A further indirect but highly relevant line comes from maternal immune activation (MIA) and prenatal inflammatory risk models. Reviews of ASD neuroinflammation consistently highlight maternal infection, immune activation, and early-life inflammatory disturbances as credible contributors to ASD risk [132]. Bilbo et al., [124] explicitly describe the cleavage of pro-IL-1β by caspase-1 via an inflammasome complex as relevant to environmentally driven maternal immune activation models of ASD. More recently, a 2025 review [133] focused specifically on the P2X7/NLRP3/IL-1β axis in maternal infection-related neurodevelopmental disorders states that MIA-induced increases in maternal plasma and fetal brain cytokines, including IL-1β, were regulated by the P2X7–NLRP3 pathway in a mouse model of ASD.

Thus, the circumstantial evidence in ASD is substantial: neuroinflammation is well supported, microglial dysfunction is plausible, IL-1 family cytokines are dysregulated, and maternal immune activation strongly overlaps with inflammasome biology.

#### 3.7.3. Animal Causal Evidence

The causal animal evidence is promising but still less consolidated than in AD or MDD. The most important mechanistic route appears to be through maternal immune activation and purinergic P2X7/NLRP3/IL-1β signaling.

Szabó et al. [133] state that, in a *mouse* model of ASD, the MIA-induced increase in maternal plasma and fetal brain cytokines was regulated by the P2X7–NLRP3 pathway, explicitly naming fetal brain IL-1β among the affected mediators. This places NLRP3 biology at a developmental stage where autism-relevant neuroimmune disruption could plausibly alter neuronal migration, neurite outgrowth, synaptic maturation, and later behavior.

A related purinergic review [134] notes that suramin, a non-selective P2X/P2Y antagonist, corrected multiple abnormalities in a maternal immune activation model showing ASD-like phenotypes, including core social deficits. This does not prove NLRP3 specifically, but it is mechanistically relevant because P2X7 receptor activation is one of the classic upstream triggers of NLRP3-mediated IL-1β processing. The purinergic rescue data strengthen the inflammasome hypothesis without being fully specific for it.

Overall, the animal literature supports a model in which prenatal inflammatory stress, purinergic signaling, IL-1β maturation, and microglial activation converge on ASD-like neurodevelopmental abnormalities. But compared with AD, the ASD field still lacks a large body of clean experiments showing that genetic deletion of NLRP3, ASC, or caspase-1, or use of a selective NLRP3 inhibitor, robustly rescues core autism-like behaviors across models.

#### 3.7.4. Major Gaps

There is a shortage of primary *human* studies directly measuring the full canonical inflammasome pathway in ASD, especially studies combining NLRP3, ASC, cleaved caspase-1, IL-1β, and IL-18 in the same cohort. Much of the *human* literature emphasizes cytokine dysregulation and glial activation rather than direct inflammasome function. ASD is profoundly heterogeneous, and immune abnormalities likely characterize only a subset of patients rather than the whole spectrum. Much of the mechanistic evidence comes from MIA and environmental risk models, which are informative but may reflect only one etiologic route into ASD. Finally, there are currently no established clinical trials in ASD specifically targeting NLRP3, ASC, caspase-1, or the downstream IL-1/IL-18 axis.

### 3.8. Cross-Disorder Synthesis

Several principles emerge from this comparison. First, NLRP3 is a recurrent convergence node for ATP-P2X7 signaling, mitochondrial ROS, NF-kB priming, and sterile danger signaling. Second, microglia remain the dominant cellular effectors, but astrocytes increasingly appear as upstream amplifiers and downstream sustainers of pathology. Third, the functional consequence of inflammasome activation differs according to disease context: mood and trauma disorders emphasize stress-related neuroimmune amplification; schizophrenia emphasizes circuit dysmaturation and synaptic pruning; AD emphasizes proteinopathy amplification; and ASD emphasizes developmental glial programming [59,77,86,112,135].

A practical hierarchy of mechanistic maturity can therefore be proposed (Table 1): AD shows the strongest evidence for NLRP3 as a core driver; MDD shows strong evidence for NLRP3 as a major stress-linked amplifier; PTSD and BD occupy an intermediate position with growing translational coherence; schizophrenia remains plausible but still indirect; and ASD is best framed as subgroup- and developmental-context dependent. This hierarchy is useful for prioritizing both biomarker development and therapeutic translation.

## 4. Mechanisms Linking Inflammasomes to Neuropsychiatric Disorders

### 4.1. Neuroinflammation

Neuroinflammation is a regulated immune response of the central nervous system (CNS) that becomes pathogenic when glial activation, cytokine release, and danger-signal sensing persist over time. Among inflammasome platforms, the NLRP3 inflammasome is the most intensively studied in the context of CNS disease [136]. NLRP3 is the most relevant to neuropsychiatric research [137], emerging less as a disorder-specific lesion than as a stress-sensitive inflammatory transducer that links danger signals, microglial activation, astrocyte responses, and neuronal dysfunction into a sustained neuroinflammatory phenotype relevant to depression, anxiety, PTSD, and, more indirectly, SZ, BD, and ASD [138,139,140]. Dong et al. [137] explicitly describe NLRP3 as recognizing stimuli including ROS and extracellular ATP, and note that stress-associated DAMPs such as ATP and heat shock proteins can induce its activation in PTSD-like conditions. This makes NLRP3 especially suitable for neuropsychiatric models, where sterile stress rather than infection is often the initiating event.

Mechanistically, NLRP3 operates through a two-step process: priming, then activation. The chronic sleep deprivation study emphasizes that these temporally distinct steps may increase susceptibility to neuropsychiatric symptoms after stress exposure [141]. This observation might explain how repeated psychosocial or metabolic stress can gradually push the CNS from adaptive immune surveillance into chronic neuroinflammatory dysregulation.

The optimal neuroinflammation model seems to include microglia (as the dominant early effector cell), astrocytes (as amplifiers involved in remodeling) and neurons (whose involvement becomes more visible under chronic or severe pathological states).

#### Physiological Surveillance Versus Pathological Neuroinflammation

Under normal conditions, microglia carry out immune surveillance and monitor the brain parenchyma. In this state, inflammasome-linked signaling is tightly constrained and compatible with tissue homeostasis. In depression-focused microglial literature, resting microglia are explicitly described as immune sentinels under physiological conditions. Pathology begins when microglia move from surveillance into a primed and persistently reactive state, with NLRP3 functioning as a molecular switch that sustains cytokine release and inflammatory amplification. In neuropsychiatric settings, this transition is driven by chronic stress, sleep deprivation, trauma, glucocorticoid excess, oxidative stress, and ATP/DAMP-rich extracellular environments.

This progression is supported by chronic sleep deprivation, where increased NLRP3 expression, cytokine production, and microglial activation accompanied anxiety phenotypes; both anxiety and neuroinflammation can be suppressed by inhibiting NLRP3 signaling [141]. Dong et al. [137] likewise show that NLRP3 activation occurs very early after traumatic stress exposure, before systemic inflammation, supporting its role as an initiating CNS inflammatory mechanism rather than a late bystander.

**Figure 3:**
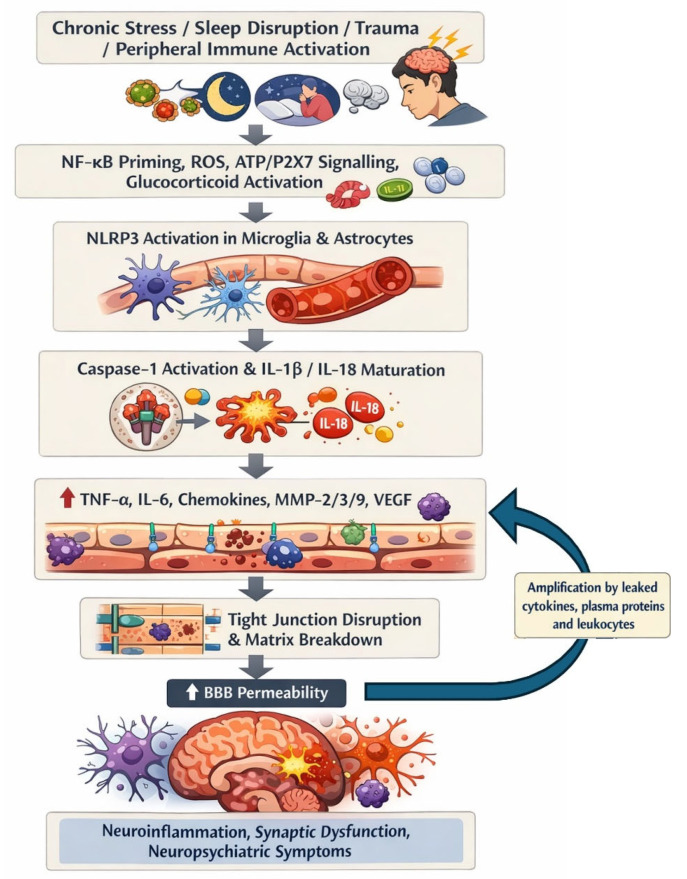
Stress-associated NLRP3 inflammasome activation promotes blood–brain barrier disruption and feed-forward neuroinflammation. ATP/P2X7—signaling axis; BBB—blood brain barrier; IL-1β—interleukin 1β; IL-18—interleukin 18; MMP—metalloproteinases; NF-KB—nuclear factor kappa B; NLRP3—nucleotide-binding domain, leucine-rich repeat family, pyrin domain containing protein 3; ROS—reactive oxygen species; TNF-α—tumor necrosis factor-alpha; VEGF—vascular endothelial growth factor.

The amplification loop involving IL-1β and IL-18 release, with IL-1β promoting leukocyte migration and cytokine cascades, and IL-18 stimulating further inflammatory amplification is important in neuropsychiatric disease; thus IL-1β and IL-18 are not merely immune markers but are also linked to persistent glial activation, altered glutamatergic signaling, oxidative stress, synaptic and dendritic dysfunction, neuronal apoptosis, and long-lasting changes in stress-sensitive brain regions such as the hippocampus, amygdala, and prefrontal cortex [1]. NLRP3 appears to convert stress biology into a cytokine program that reshapes neural circuits (Figure 3).

### 4.2. Blood–Brain Barrier Disruption

The BBB as a neuroimmune interface may be compromised by peripheral inflammasome activation, which may allow inflammatory mediators to enter the brain. NLRP3 is not only a glial inflammatory amplifier; it is also a vascular destabilizer. In neuropsychiatric disorders, especially those with a strong inflammatory or stress-related component, NLRP3-linked signaling may help convert systemic stress and peripheral inflammation into central pathology by weakening BBB integrity, altering endothelial signaling, changing cytokine transport dynamics, and promoting leukocyte entry into the CNS. BBB dysfunction can expose the brain to peripheral cytokines, plasma proteins, danger signals, and immune cells that further reinforce glial activation and circuit dysfunction. Despite recent proof of the connection between NLRP3-driven neuroinflammation and BBB breakdown, only a few direct proofs within each psychiatric diagnosis exists [59,142].

The BBB is formed primarily by brain microvascular endothelial cells, supported by pericytes, astrocytic endfeet, the basement membrane, and close interactions with microglia and neurons. Endothelial cells are linked by tight junctions (TJs) and adherens junctions (AJs) that restrict paracellular flux and maintain selective transport. The main TJ proteins repeatedly highlighted across BBB literature are claudin-5, occludin, junctional adhesion molecules (JAMs), and the scaffolding proteins ZO-1, ZO-2, and ZO-3 [143,144,145]. Pericytes support the endothelial barrier phenotype and induce synthesis of TJ proteins, while astrocytes regulate barrier integrity through trophic and morphogenetic signaling pathways [146].

From a mechanistic perspective, neuroinflammation-associated blood–brain barrier failure comprises two principal components: physical barrier impairment and cytokine passage across the endothelial interface.

Physical barrier impairment generally occurs through four interacting routes, all involving NLRP3 signaling:

(a) Tight-junction disassembly or downregulation
(b) Enhanced endothelial transcytosis
(c) Basement membrane/extracellular matrix degradation
(d) Leukocyte adhesion and transmigration

NLRP3 activation products are highly relevant to BBB dysfunction, as IL-1β, IL-18, TNF-α, ROS, chemokines, and MMPs directly affect endothelial junctional stability and vascular permeability [1,59,146]. Although microglia are the main early effector cell in many CNS inflammatory states, NLRP3-related activity has been reported in astrocytes and also in vascular-associated compartments, including endothelial-relevant neurovascular contexts. In practice, even when microglia are the initiating source, the downstream mediators they release act directly on endothelial cells and astrocytes to destabilize the BBB [1,147].

(a) *Inflammasome–TJ interferences* are prone to the following mechanistic sequence: NLRP3 activation → IL-1β/IL-18/TNF-α/ROS/MMP induction → reduced expression or altered localization of claudin-5, occludin, ZO-1 → increased paracellular permeability.

Claudin-5, dominant in BBB TJs (tight junctions), is a major determinant of paracellular sealing. Reduced claudin-5 expression is repeatedly associated with barrier leakiness. Inflammatory and oxidative conditions downregulate claudin-5 in endothelial cells and increase permeability [143,145]. In a stroke model, MCC950 pharmacological NLRP3 inhibition partially restored claudin-5 and ZO-1 (zonula occludens) in enriched cerebral microvessels while reducing extravasation, directly linking NLRP3 activity to TJ loss and barrier dysfunction [148].

In a neurovascular AD-related context, IL-1β released from activated microglia increased BBB permeability and downregulated ZO-1, occludin, and claudin-5, while suppressing astrocytic sonic hedgehog support for BBB integrity [147].

Occludin is a critical transmembrane TJ protein whose loss correlates with increased paracellular permeability. Occludin expression and phosphorylation status are closely tied to barrier integrity, and inflammatory injury often reduces occludin abundance or redistributes it away from junctional membranes [145,146]. IL-1β and TNF-α both contribute to occludin downregulation in endothelial cells, thereby increasing BBB permeability [149].

ZO-1 functions as a cytoplasmic scaffold linking transmembrane TJ proteins to the actin cytoskeleton. Downregulation or junctional redistribution of ZO-1 destabilizes the entire barrier architecture. Reduced ZO-1 is a marker of barrier opening in inflammatory and oxidative conditions [143,145]. Again, NLRP3 inhibition in vivo preserved ZO-1 in cerebral microvessels, which is strong evidence that inflammasome signaling contributes to structural TJ disassembly rather than simply correlating with it [148].

(b) *Endothelial inflammasome activation and endothelial vulnerability* may rely on the following mechanistic sequence: stress/ROS/inflammatory ligands → priming of endothelial dysfunction → plus microglial–astrocytic inflammasome output that exposes endothelium to IL-1β, TNF-α, chemokines, MMPs → drives junctional collapse and BBB leakiness.

Endothelial cells are highly sensitive to ROS, TLR activation, Aβ/RAGE signaling, cytokines, and mitochondrial dysfunction, all of which can converge on inflammasome-relevant pathways and barrier destabilization [145,147]. In mouse brain endothelial cells, Aβ42 binding to RAGE downregulated occludin and ZO-1 and increased endothelial barrier permeability; also, increased MMP expression induces BBB permeability as microglial IL-1β worsens endothelial barrier loss [147]. Endothelial cells are active responders to inflammasome-associated inflammatory mediators, but also endothelial NLRP3 assembly is not always directly measured in psychiatric models. Oxidative stress itself can regulate endothelial barrier genes. ROS increase expression of TLRs in BBB endothelial cells, causing downregulation of occludin and claudin-5, while hypoxia reduces claudin-5, occludin, and ZO-1 [145]. Since ROS are also major upstream activators of NLRP3, endothelial stress and inflammasome activation likely reinforce one another.

(c) *Basement membrane/extracellular matrix degradation*. MMPs are a crucial bridge between inflammasome signaling and structural BBB failure. Even if MMPs are mainly described in stroke, hemorrhage, and neurodegeneration [150], they are highly relevant to neuropsychiatric disorders, as the same inflammatory mediators (IL-1β, TNF-α, ROS, NF-κB signaling) are repeatedly implicated in affective and psychotic disorders with neuroinflammatory signatures. Thus, MMP-mediated BBB injury is a plausible downstream executor of psychiatric inflammasome biology, especially in treatment-resistant depression, PTSD, bipolar disorder with inflammatory activation, and psychosis-associated vascular dysfunction. MMP-9 is one of the most important BBB-disruptive proteases. It degrades tight-junction proteins and extracellular matrix components, weakening the neurovascular unit. Resolvin D1 reduces NLRP3, cleaved caspase-1, IL-1β, and MMP-9, while simultaneously increasing ZO-1, occludin, and claudin and improving BBB integrity [151]. The mechanistic chain can be described as: NLRP3/NF-κB signaling up → MMP-9 up → tight-junction degradation → BBB disruption. MMP-2 degrades occludin and claudin-5, while MMP-3 increases BBB permeability through ERK-linked disruption of ZO-1, claudin-5, and occludin [145]. In inflammatory CNS conditions, astrocytes release VEGF and MMPs that degrade tight-junction proteins and extracellular matrix, causing BBB disruption [143].
(d) *Leukocyte adhesion and transmigration*: this route is critical because NLRP3 signaling not only weakens the BBB structurally but also converts the brain endothelium into an activated inflammatory interface that recruits circulating immune cells. Under physiological conditions, BBB endothelial cells express very low levels of adhesion molecules, which helps prevent leukocyte entry into the CNS [152]. By contrast, in a BBB endothelial model, inflammatory injury engages caspase-1 and responds by regulating ICAM-1 and E-selectin, encouraging leukocyte–endothelial adhesion [153]. Mechanistically, NLRP3 generates downstream mediators (IL-1β, IL-18, TNF-α, CCL2 and related chemokines, ROS/NF-κB-linked endothelial activation) that induce a pro-adhesive endothelial phenotype, allowing circulating leukocytes to tether, roll, firmly adhere, and then cross the BBB [154]. The leukocyte transmigration component is more often inferred from mechanistic overlap than directly measured in *human* psychiatric cohorts.

Following the events induced by BBB disruption, cellular death is accelerated by induced ischemia, which also causes additional inflammatory damage to the surrounding brain tissue [32].

#### Cytokine Passage Across the Endothelial Interface may Occur by Various Ways

Active transport and humoral routes [1] describe the passage of pro-inflammatory cytokines into the brain through active transporters or action at regions lacking a classical BBB, and so peripheral inflammation can influence the brain even before gross BBB rupture.

Paracellular leak occurs on weakened tight junctions due to claudin-5, occludin, and ZO-1 downregulation, and cytokines and plasma proteins gain greater access to the brain parenchyma. BBB dysfunction engages altered endothelial vesicular trafficking and adsorptive-mediated transcytosis as a pathway by which positively charged plasma proteins such as albumin bind endothelial surfaces and are endocytosed into the brain [143].

Inflammatory states can alter transporter expression, especially ABC transporters such as ABCB1/P-gp, ABCC/MRPs, and ABCG2/BCRP, which regulate extrusion of xenobiotics and many small molecules [146] and can thereby reshape brain exposure to circulating molecules. In psychiatric disease, this may influence both inflammatory burden and psychotropic pharmacokinetics.

Systemic inflammation can affect the brain through increased BBB permeability creating a feed-forward loop: NLRP3 → IL-1β/IL-18 → Th17-skewing/IL-17 signaling → tight junction loss → more leukocyte entry/cytokine flux; this loop is highly relevant to psychiatric disorders with peripheral immune activation [59].

BBB disruption also involves non-endothelial cells. Under inflammatory conditions, astrocytes may switch from barrier-supportive cells to barrier-destabilizing amplifiers by releasing VEGF and MMPs to degrade claudin-5 and occludin and damage the extracellular matrix [143]. Microglial IL-1β suppresses astrocytic sonic hedgehog support of BBB integrity and stimulates astrocytes to produce chemokines such as CCL2 (C-C Motif Chemokine Ligand 2), CCL20 (C-C Motif Chemokine Ligand 2), and CXCL2 (C-X-C Motif Chemokine Ligand 2), which exacerbate migration and BBB disruption [147]. Pericytes induce TJ protein synthesis and maintain the endothelial barrier phenotype. Pericytes support synthesis of occludin, claudin-1, ZO-1, and ZO-2 via proangiogenic factors, while pericyte–endothelial crosstalk stabilizes the BBB [146]. Therefore, inflammasome-related injury that perturbs pericyte signaling can indirectly destabilize the barrier even without direct endothelial NLRP3 assembly. Vessel-associated microglia can initially maintain BBB integrity, but inflammatory microglial cytokines later promote permeability, including IL-1β-mediated downregulation of TJ proteins [149] with microglial dysfunction contributing to persistent BBB disturbance in neuropsychiatric disorders [139].

### 4.3. Microglial Overactivation

Microglia play a crucial role in maintaining the health of the central nervous system through synaptic pruning, debris clearance, and pathogen elimination; however, sustained microglial inflammation can lead to neuronal damage [155]. In neurological diseases, neuroinflammation is exacerbated by microglial overactivation, which triggers the inflammasome, a multi-protein complex that releases pro-inflammatory cytokines like IL-1 and IL-18. This overactivation creates a self-perpetuating cycle, where chronic inflammation drives neuronal damage and disease progression. Microglia’s inflammasomes can lead to chronic neuroinflammation and neuronal damage.

Perineuronal nets (PNNs) are netlike sheaths surrounding the somas of a subset of neurons, especially parvalbumin-positive, GABA-ergic fast-spiking interneurons [156,157]. Because PNNs stabilize synapses, their formation and maintenance are essential for memory retention. Additionally, because PNN components have antioxidant qualities, they protect neuron somas from external reactive oxygen species [157]. Microglial activation has been demonstrated to induce degradation of PNNs of primary neurons in vitro and in mammalian brains in vivo [158,159].

Tarakcioglu et al. showed that soluble molecules, such as proteins, released from NLRP3-activated microglia reduce PNN-positive neurons in a microglia–neuron coculture model. This suggests that in acute microglial activation-induced neuroinflammation, drugs directly targeting NLRP3 could prevent PNN degradation. Without relying on broad anti-inflammatory drugs like NSAIDs or steroids, which can cause side effects at high doses, this approach could prevent PNNs from inflammation-associated damage [155].

### 4.4. HPA Axis Dysregulation

Chronic stress disrupts the hypothalamic–pituitary–adrenal (HPA) axis, and this endocrine dysregulation represents an upstream stress-response abnormality rather than an inflammatory mechanism per se. Under physiological conditions, activation of the HPA axis culminates in glucocorticoid release, which normally constrains immune activation through negative feedback. However, prolonged stress may induce persistent HPA-axis activation, impaired glucocorticoid receptor signaling, and loss of negative feedback, resulting in sustained cortisol dysregulation and reduced anti-inflammatory control. In this framework, HPA-axis dysfunction should be understood as an initiating neuroendocrine event that increases vulnerability to downstream immune and neuroinflammatory activation rather than as a direct mediator of excitotoxic injury itself [1,160].

A growing body of evidence indicates that the consequences of chronic stress extend beyond endocrine dysregulation and include a second, downstream inflammatory phase characterized by microglial activation, induction of the NLRP3 inflammasome, and increased expression of pro-inflammatory cytokines. Thus, HPA-axis dysfunction and inflammasome activation are closely linked but mechanistically distinct: the former reflects maladaptive stress-hormone signaling, whereas the latter reflects activation of innate immune pathways within the CNS. Stress signals reach the brain through both neural pathways, including vagal afferents, and humoral pathways, involving cytokine transport across the blood–brain barrier or signaling at circumventricular regions lacking a fully developed barrier [1].

Within this downstream inflammatory phase, pro-inflammatory cytokines bind to receptors on endothelial cells, astrocytes, and microglia and activate transcriptional cascades such as NF-κB and MAPK, which promote further cytokine production and inflammasome priming. In parallel, endogenous DAMPs activate TLRs and stimulate assembly of the NLRP3 inflammasome, thereby increasing caspase-1-dependent maturation of IL-1β and IL-18 [1,161].

A third, downstream neurotoxic phase may then emerge from this inflammatory milieu. Microglia, acting as central modulators of the inflammatory response, alter cytokine output in the context of chronic stress and HPA-axis dysregulation, resulting in elevated IL-1β and IL-18 production linked to NLRP3 activation. These cytokines do not merely signal inflammation; they also interfere with neuronal and astrocytic homeostasis. Elevated hippocampal IL-1β is associated with impaired neurogenesis, disruption of long-term potentiation (LTP), and altered memory processing, while increased IL-18 in limbic regions has been associated with anxiety- and depression-related behavioral changes [162].

Importantly, excitotoxicity should be considered a downstream consequence of cytokine-driven glial–neuronal dysregulation, not a direct manifestation of HPA-axis dysfunction. Inflammatory mediators such as IL-1β can enhance presynaptic glutamate release and impair astrocytic glutamate clearance, including reduced activity of transporters such as GLT-1, thereby promoting excessive stimulation of NMDA receptors and increasing the risk of excitotoxic damage. Accordingly, the mechanistic sequence is best conceptualized as: chronic stress → HPA-axis dysregulation → loss of glucocorticoid-mediated immune restraint → microglial/inflammasome activation → cytokine amplification → glutamatergic dysregulation and excitotoxicity, rather than as a single undifferentiated process [1].

## 5. Inflammasomes as Therapeutic Targets in Neuropsychiatric Disorders

Neuropsychiatric disorders often feature neuroinflammation in which the NLRP3 inflammasome plays a pivotal role. Therapeutic strategies to modulate NLRP3 signaling fall into several broad categories, each with distinct disease targets within the neuropsychiatric spectrum and varying stages of clinical development. Hence, the current treatment options have moved toward CNS-directed, brain-penetrant small molecules and pathway-level inhibitors (i.e., upstream purinergic/ATP signaling and IL-1 blockade), targeting (but not being limited to) inflammasomes, mainly NLRP3. However, no marketed drug that specifically targets NLRP3 is yet approved, and the need for CNS penetration is widely emphasized as a key translational constraint for neurological indications [16].

### 5.1. Direct NLRP3 Inhibitors

Direct blockade of NLRP3 prevents assembly and activation of Caspase-1 and subsequent IL-1β/IL-18 maturation, addressing a proximal node in brain inflammasome signaling implicated in neuroinflammation and neuronal dysfunction [163,164]. Certain compounds that function as specific inhibitors of the NLRP3 inflammasome are currently in advanced stages of clinical development [165].

Direct NLRP3 inhibitors address neuroinflammatory components of Parkinson’s disease (PD), Alzheimer’s disease (AD), and neurodevelopmental/psychiatric conditions with associated microglial activation and cognitive or affective symptoms. Preclinical PD/AD models show reduced inflammasome activity and improved outcomes with NLRP3 inhibitors or downstream readouts [163,166]. Comparative data regarding direct NLRP3 inhibitors are displayed in Table 2.

### 5.2. Upstream Pathway Modulation (mtROS, mtDNA, Mitophagy, Mitochondria–NLRP3 Interfaces, Antioxidants)

Mitochondrial dysfunction and mtROS/mtDNA leakage are upstream drivers of NLRP3 activation. Therapeutic strategies that preserve mitochondrial integrity, reduce mtROS, or enhance mitophagy can downstream suppress NLRP3 signaling and attenuate neuroinflammation [163].

Mitochondrial pathway modulation addresses PD and other neurodegenerative diseases with mitochondrial dysfunction; mood and cognitive disorders where neuroinflammation is implicated (e.g., sleep deprivation-related anxiety, aging-related cognitive decline) may benefit from mitochondrial stabilization approaches [187,188].

Therapeutic attenuation of neuroinflammation can be approached upstream at the level of mitochondrial quality control [189]. Interventions that preserve inner-membrane integrity, reduce mitochondrial ROS production, and enhance mitophagic clearance of damaged mitochondria are expected to limit DAMP release, inflammasome activation, and maladaptive microglial polarization [190]. In translational terms, this framework is represented by MitoQ for mtROS suppression, elamipretide/SS-31 for mitochondrial membrane stabilization, and urolithin A as the leading *human* mitophagy inducer [191], with spermidine and NAD+ precursors as supportive mitophagy-oriented candidates. Comparative data regarding upstream NLRP3 pathway modulators are displayed in Table 3.

### 5.3. Indirect Inflammasome Modulation via Metabolic Reprogramming

A special way to modulate NLRP3 activation is to address cellular metabolism, including glycolysis and mitochondrial pyruvate flux. Systemic metabolic effects and compensatory pathways require careful dosing and monitoring, but integration with CNS-specific readouts is essential. Shifting metabolism toward oxidative phosphorylation and limiting lactic acid fermentation can dampen mtROS and inflammasome assembly, offering a metabolic angle to neuroinflammation control [190].

Indirect inflammasome modulation can be applied in mood disorders and cognitive impairment where metabolic dysregulation and neuroinflammation co-occur; PD and aging-related neuroinflammation may also respond to metabolic modulators that suppress NLRP3 instead of direct blockade [163,236]. Comparative data regarding indirect NLRP3 pathway modulators are displayed in Table 4.

### 5.4. Upstream Inflammatory Priming/ER-Golgi and Trafficking Targets

Some alternative intervention points to impede inflammasome formation without entirely shutting downthe immune response are represented by signals that prime or facilitate NLRP3 assembly, including ER–Golgi trafficking and NEK7 docking [164]. This approach can address CNS inflammatory states in MS and neurodegenerative conditions where microglial NLRP3 activation is prominent; preclinical data suggest that blocking trafficking steps reduces CNS inflammasome activation and neuroinflammation [251]. It is important to explore specificity to CNS secretory pathways and to avoid broad suppression of critical ER-Golgi functions in neurons; safety profiles also require thorough CNS-focused toxicology. Comparative data regarding upstream inflammatory priming/ER-Golgi and trafficking targets are displayed in Table 5.

### 5.5. Downstream Cytokine Signaling Blockade (IL-1/IL-18 Axis)

IL-1β and IL-18 are major effector cytokines downstream of NLRP3; blocking IL-1 signaling (e.g., IL-1 receptor antagonists like anakinra or IL-1β antibodies like canakinumab) can mitigate inflammasome-driven pathology in neuroinflammation and associated neuropsychiatric symptoms, while sometimes bypassing upstream mitochondrial triggers [260].

The current approach can be used in mood disorders with inflammatory signatures, some forms of psychosis and behavioral disturbances linked to IL-1β/IL-18; stroke-related cognitive impairment and perioperative neuroinflammation contexts have shown benefit from IL-1 blockade in CNS injuries [260].

### 5.6. Antidepressants with Anti-Inflammatory Effect

Antidepressant drugs may have anti-inflammatory and autophagic effects. Tricyclic antidepressants, including citalopram, imipramine, and clomipramine, have anti-inflammatory effects by inhibiting the release of IL-6, IL-1, and TNF in *human* monocytes [261]. Treatment with antidepressants, such as fluoxetine, paroxetine, mianserin, mirtazapine, venlafaxine, desvenlafaxine, amitriptyline, imipramine, and agomelatine, has been shown in clinical studies to induce autophagy and reduce the expression of NLRP3 inflammasome components and inflammatory cytokines, such as IL-1β and IL-18 [262].

### 5.7. Orexinergic System and Inhibitors

In addition to direct inflammasome-directed approaches, emerging evidence suggests that the orexinergic system is relevant to neuropsychiatric disorders characterized by hyperarousal, sleep disruption, anxiety, and maladaptive fear processing, including PTSD. Orexin/hypocretin circuits regulate arousal, REM sleep, autonomic activation, motivation, and emotional memory, and recent reviews highlight their broader implication in PTSD, panic, phobia, anxiety, depression, and addiction [263,264]. Consistent with this, recent preclinical work has demonstrated a role for hypocretin signaling in PTSD-like behaviors, including enhanced fear memory, anxiety-like behavior, and altered sleep after prolonged stress exposure [265]. From a therapeutic perspective, suvorexant, a dual orexin receptor antagonist (DORA) approved for insomnia, has attracted interest as a translational candidate. *Human* experimental evidence now includes a double-blind randomized pilot trial testing suvorexant on fear extinction recall, a PTSD-relevant process, although no significant direct improvement in extinction recall was observed [266]. Clinically, suvorexant has also been evaluated in trauma-related insomnia, including individuals with and without PTSD, supporting its feasibility in trauma-exposed populations [267]. Together, these data suggest that orexin antagonism may represent a promising adjunctive strategy in neuropsychiatric disorders, particularly where sleep disruption, hyperarousal, and extinction-related abnormalities interact with neuroinflammatory mechanisms.

## 6. Conclusions

Briefly, this review was conducted as a narrative, mechanistic, integrative review focused on the NLRP3 inflammasome across neuropsychiatric and neurodegenerative disorders, rather than as a formal systematic review. The literature survey was performed using the major biomedical databases PubMed/MEDLINE, Scopus, and Web of Science. The search primarily covered the period from 2010 to March 2026, while older seminal studies were included when necessary to preserve mechanistic and historical context. The main search terms combined “NLRP3 inflammasome”, “inflammasome”, “neuroinflammation”, “neuropsychiatric disorders”, “depression”, “bipolar disorder”, “schizophrenia”, “PTSD”, “autism spectrum disorder”, “Alzheimer’s disease”, “microglia”, “astrocytes”, “blood–brain barrier”, “mitochondrial dysfunction”, and “therapeutic targets/therapy”. In general, we prioritized peer-reviewed English-language articles with direct relevance to the mechanistic role of NLRP3 signaling, including original experimental studies, translational *human* studies, animal studies, and high-quality reviews. Preference was given to studies providing direct mechanistic, cellular, molecular, or therapeutic insight, whereas papers with only marginal relevance to the review focus were not retained.

Collectively, the evidence reviewed here supports a contemporary neuroimmune framework in which the NLRP3 inflammasome functions as a stress-responsive molecular integrator linking danger signaling, glial activation, mitochondrial dysfunction, blood–brain barrier vulnerability, and maladaptive circuit remodeling across a broad spectrum of neuropsychiatric and neurodegenerative disorders. Rather than representing a uniform lesion across diagnoses, NLRP3 appears to occupy different pathogenic positions depending on disease context, ranging from a relatively central and causally mature driver in AD to a more conditional, amplificatory, or subgroup-dependent role in major depressive disorder, bipolar disorder, post-traumatic stress disorder, schizophrenia, and autism spectrum disorder.

A major conceptual advance emerging from this literature is that neuropsychiatric disorders can no longer be interpreted adequately through a single-neurotransmitter lens. Instead, they are better understood as disorders of networked biological instability, in which stress physiology, glial reactivity, sleep–circadian disruption, neurovascular dysfunction, innate immune priming, and impaired plasticity become mutually reinforcing. Within this architecture, NLRP3 is not merely an inflammatory marker but a plausible conversion point through which sterile stress is translated into sustained cytokine signaling, pyroptotic reinforcement, synaptic dysregulation, and long-term behavioral dysfunction.

The review also highlights that the strength of evidence is not equivalent across disorders. Alzheimer’s disease currently offers the most coherent and causally persuasive model, supported by direct *human* evidence, strong mechanistic integration with amyloid-β and tau biology, and robust animal rescue data following NLRP3 or caspase-1 suppression. Major depressive disorder follows as the most compelling psychiatric context, where chronic stress, ATP–P2X7 signaling, ROS, and kynurenine pathway dysregulation converge on a well-developed inflammasome model. Bipolar disorder and PTSD occupy an intermediate position, with biologically coherent but still incomplete translational evidence, whereas schizophrenia and ASD remain better supported by indirect, developmental, cellular, or subgroup-specific findings than by definitive proof of universal NLRP3 centrality.

An additional strength of the current framework is the integration of BBB dysfunction and neurovascular injury into inflammasome biology. The available evidence suggests that NLRP3 signaling does not only acts within microglia but can also reshape the CNS inflammatory milieu by destabilizing endothelial junctions, altering cytokine trafficking, enhancing leukocyte recruitment, and amplifying feed-forward interactions between microglia, astrocytes, and vascular compartments. This vascular dimension is especially relevant because it provides a mechanistic route by which peripheral inflammation may be translated into central pathology even before overt structural injury becomes visible.

Finally, the review supports the view that NLRP3 should be regarded less as an isolated inflammasome and more as a systems-level inflammatory node embedded within mitochondrial quality control, purinergic signaling, autophagy, orexin-linked arousal dysregulation, and stress-endocrine circuitry. This systems interpretation is essential if inflammasome biology is to be translated meaningfully into biomarker development and therapy selection in complex brain disorders.

## 7. Future Directions

First, the field now needs to move beyond descriptive inflammation and toward mechanistically resolved patient stratification. A major limitation across neuropsychiatric research is the continued treatment of diagnostic categories as biologically homogeneous entities. The evidence reviewed here instead favors the existence of inflammatory or immunometabolic endophenotypes, in which NLRP3-related signaling is likely to be relevant only in subsets of patients. Future studies should therefore combine peripheral cytokine profiling, inflammasome-related transcripts and proteins, mitochondrial stress markers, neuroimaging, sleep/circadian phenotyping, and clinical symptom dimensions to identify the populations most likely to exhibit inflammasome-dependent pathology.

Second, there is a pressing need for more direct *human* evidence, particularly at the central level. In many psychiatric conditions, the current literature still relies heavily on peripheral blood markers, indirect inflammatory signatures, or postmortem tissue with substantial confounding. The next-generation of translational studies should prioritize direct measurement of NLRP3 assembly, ASC speck formation, cleaved caspase-1, mature IL-1β/IL-18, and pyroptosis-related markers in better-characterized *human* biospecimens. Parallel development of imaging-compatible or fluid-based biomarkers capable of reflecting inflammasome activity in vivo would represent a major advance for the field.

Third, future work should address cell-type and compartment specificity more rigorously. Although microglia remain the dominant effector cells in most NLRP3 models, the data reviewed here strongly suggest that astrocytes, endothelial cells, pericytes, peripheral myeloid cells, and neurons also participate in inflammasome-related signaling cascades, either as upstream amplifiers, downstream responders, or barrier-modifying intermediates. Resolving the temporal and spatial sequence of these interactions will be essential for understanding why similar upstream stressors lead to distinct clinical phenotypes across disorders.

Fourth, the most promising therapeutic future likely lies not in a single class of inhibitors, but in a tiered intervention strategy. The review indicates that NLRP3 can be targeted at multiple levels: direct inflammasome blockade; upstream modulation of mtROS, mitophagy, and mitochondrial integrity; indirect metabolic reprogramming; inhibition of priming and trafficking pathways; downstream IL-1/IL-18 blockade; and repurposing of agents with secondary anti-inflammatory effects, including selected antidepressants and orexin-related interventions. This layered model is particularly attractive because it allows therapy to be adapted to disease architecture: direct inhibition may be more appropriate in AD and severe inflammatory states, whereas upstream mitochondrial or metabolic modulation may be better suited to mood disorders or trauma-related phenotypes.

Fifth, the interaction between sleep–wake regulation, orexinergic signaling, and inflammasome activity deserves far greater attention. The integration proposed in this review suggests that arousal instability, circadian disruption, and chronic sleep fragmentation may not merely accompany neuropsychiatric disease, but may actively lower the threshold for microglial priming, NF-κB activation, mitochondrial stress, and subsequent NLRP3 engagement. This opens an important avenue in which sleep biology is not peripheral to neuroinflammation, but one of its most clinically actionable regulators.

Finally, a central translational challenge remains: how to bridge strong preclinical causality with clinically meaningful therapeutic efficacy. This gap is especially visible in disorders such as AD, where causal animal data are compelling but *human* validation is still incomplete. Progress will likely depend on biomarker-guided trial design, earlier intervention windows, and selection of patients whose disease biology genuinely converges on inflammasome-linked pathways. In this respect, the future of the field may depend less on asking whether NLRP3 is involved in a disorder in general, and more on identifying when, where, and in whom inflammasome signaling becomes pathologically decisive.

Overall, the most defensible perspective is that the NLRP3 inflammasome has emerged as one of the most biologically coherent links between stress biology, neuroimmune activation, and circuit dysfunction in brain disease, but its translational value will depend on precision phenotyping, mechanistic biomarkers, and disease-contextualized therapeutic deployment. In that sense, the inflammasome is unlikely to become a universal explanation for neuropsychiatric pathology; rather, it is poised to become a high-value stratification and intervention axis within a broader systems–neuroscience model of mental and neurodegenerative disorders.

## Figures and Tables

**Table 1:** Comparative mechanistic profile of NLRP3 inflammasome signaling across PTSD, MDD, BD, SZ, AD, and ASD. NLRP3, nucleotide-binding oligomerization domain-like receptor family pyrin domain containing 3; PTSD, post-traumatic stress disorder; MDD, major depressive disorder; BD, bipolar disorder; SZ, schizophrenia; AD, Alz-heimer’s disease; ASD, autism spectrum disorder; ATP, adenosine triphosphate; P2X7, purinergic receptor P2X7; K^+^, potassium; NF-κB, nuclear factor kappa B; ROS, reactive oxygen species; TXNIP, thioredoxin-interacting protein; IL-1β, interleukin-1 beta; IL-18, interleukin-18; TLR2/4, Toll-like receptor 2/4; TLR4, Toll-like receptor 4; RAGE, receptor for advanced glycation end products; Ca^2+^, calcium; MAM, mitochondria-associated membranes; DAMPs, damage-associated molecular patterns; HMGB1, high mobility group box 1; HPA axis, hypothalamic–pituitary–adrenal axis; Aβ, amyloid-beta; ASC, apoptosis-associated speck-like protein containing a CARD; CNS, central nervous system; E/I, excitatory/inhibitory.

Disorder	Main Upstream Triggers	Dominant NLRP3-Linked Mechanism	Principal Downstream Pathology	Evidence for NLRP3 Centrality	Translational Note
MDD	Chronic stress, glucocorticoid disequilibrium, extracellular ATP, ROS, TXNIP, TLR2/4 priming	ATP-P2X7-K^+^ efflux, NF-kB priming, ROS/TXNIP, IL-1β/IL-18, pyroptotic reinforcement	Impaired neuroplasticity, kynurenine shift, glutamatergic dysregulation, microglia–astrocyte amplification	High–moderate	Best suited to biomarker-defined inflammatory subgroups; indirect and direct NLRP3 modulation are plausible
BD	Mitochondrial dysfunction, ROS, altered Ca^2+^ handling, metabolic stress, MAM dysregulation, ATP/P2X7	MAM-localized NLRP3 assembly linking bioenergetic failure to IL-1-family signaling	Episode sensitization, white matter/connectivity dysfunction, cognitive burden, cardiometabolic coupling	Moderate	Promising immunometabolic target, especially in patients with high inflammatory–metabolic burden
SZ	Neuronal DAMPs, HMGB1, mitochondrial stress, ROS, TLR4/NF-kB priming, complement-linked activation	NF-kB-linked upregulation of NLRP2/NLRP3 integrated with complement-mediated pruning biology	Excessive or mistimed synaptic pruning, reduced presynaptic density, dysconnectivity	Moderate–low	Most relevant as a circuit-modifying immune pathway rather than a standalone disease engine
PTSD	Trauma-related glutamatergic stress, astrocytic ATP release, P2X7 activation, ROS, HMGB1-TLR4-RAGE signaling, HPA-axis dysregulation, mitochondrial stress	Stress-induced ATP-P2X7 and HMGB1-TLR4-RAGE priming lower threshold for NLRP3 activation in fear-memory circuits	Persistent neuroinflammation, extinction deficits, synaptic remodeling, hyperarousal-linked circuit sensitization	Moderate	Mechanistically closest to MDD, but more tightly centered on trauma-encoded fear circuitry
AD	Aβ uptake/fibrils, tau species, lysosomal stress, ROS, impaired autophagy/mitophagy, microglial risk-gene pathways	Aβ/tau-driven NLRP3 activation, caspase-1 signaling, ASC speck release and feed-forward amyloid seeding	Reduced Aβ clearance, enhanced tau pathology, synapse loss, pyroptotic amplification, chronic neurodegeneration	Very high	Most compelling CNS setting for direct NLRP3 inhibition
ASD	Maternal immune activation, prenatal stress, gut-immune dysregulation, developmental cytokine skewing, genetic susceptibility	Context-dependent inflammasome engagement within altered glial maturation and pruning biology	Synaptic pruning defects, E/I imbalance, abnormal circuit formation, persistent developmental neuroimmune bias	Low–moderate	Best framed as subgroup-specific and developmentally contingent rather than universally central

**Table 2:** Direct NLRP3 inhibitors in neuropsychiatric disease. AD—Alzheimer’s disease; ALS—Amyotrophic lateral sclerosis; BBB—Blood–brain barrier; CNS—Central nervous system; IL-1β—Interleukin-1 beta; IL-18—Interleukin-18; NACHT—Nucleotide-binding and oligomerization domain found in NAIP, CIITA, HET-E, and TP1; NLRP3—NLR family pyrin domain containing 3. Compound/development codes appearing in the table: DFV890, IZD334/Somalix, MCC950, NT-0167, NT-0249, NT-0796, NDT-1795, OLT1177, ZYIL1.

**Dapansutrile (OLT1177)**			
Mechanism—Direct, oral small-molecule NLRP3 inhibitor; reported to bind the NLRP3 NACHT domain, block inflammasome assembly/caspase-1 activation, and reduce IL-1β/IL-18 maturation [167,168].			
Neuroinflammation	Preclinical evidence	*Human* trials	Translational maturity
Strong fit for neuroinflammation as NLRP3 is a central mediator of microglial IL-1β signaling, pyroptosis, and sterile CNS inflammation [16,169]	Best CNS-anchored preclinical data is the EAE/MS model, where dapansutrile ameliorated disease pathogenesis [167]. Efficacy in mouse AD model [170].	Phase I and II clinical trials for oral and topic administration showed safety [171,172] and promises for promise in reducing neuro-inflammation in the context of neuro-degenerative diseases. Phase 1B randomized, double-blind repeat-dose safety/PD study in systolic heart failure, supporting clinical tolerability and systemic pharmacodynamic development, though not in CNS disease [173].	Highest current maturity among direct NLRP3 inhibitors. Real *human* trial footprint plus CNS-relevant preclinical evidence, but no established neurologic efficacy trial yet [167,173].
**Inzomelid**			
Mechanism—Direct, oral NLRP3 inhibitor from the newer clinical wave of small molecules; BBB penetrant [168,174,175].			
Neuroinflammation	Preclinical evidence	*Human* trials	Translational maturity
High theoretical relevance for neuroinflammation, especially for selective upstream blockade of IL-1β/IL-18 processing [16,169].	Mostly supported indirectly here through review literature summarizing its emergence as a clinical-stage NLRP3 program rather than a specific standout CNS paper in this search set [168,174].	No primary clinical trial data, but recent reviews place inzomelid among the compounds in clinical development despite 2 clinical trials withdrawal [168,174]. Phase 1 clinical trials for PD, AD and ALS [176], and then withdrawn to develop another derivative—selnoflast. Selnoflast displayed few adverse effects in a Phase1b study in 2021 [116]; a second Phase 1b trial on 72 patients was started in 2022 and ended in 2024 but with no published results. Somalix (IZD334), a peripherally restricted candidate successfully passed Phase I trials and was scheduled for possible phase II development.	Moderate translational maturity. Clearly beyond purely academic chemistry, but based on this search the published *human* clinical evidence is still less visible than dapansutrile [168,174,176].
**DFV890**			
Mechanism—Direct NLRP3 inhibitor in clinical development [174,177].			
Neuroinflammation	Preclinical evidence	*Human* trials	Translational maturity
Same strong mechanistic rationale as other selective NLRP3 inhibitors for blocking inflammasome-driven innate immune activation that can propagate CNS injury [177,178].	Review-level translational summaries rather than a single defining CNS efficacy paper [178,179].	DFV890 is consistently listed in reviews as a clinical-development NLRP3 inhibitor [177,179].	Moderate translational maturity. Clearly a real translational program, but mostly pipeline/review-based rather than definitive *human* outcome data.
**MCC950**			
Mechanism—Prototype direct NLRP3 inhibitor, widely used as a benchmark preclinical tool compound; binds NLRP3 and blocks inflammasome activation [168,180].			
Neuroinflammation	Preclinical evidence	*Human* trials	Translational maturity
Very high neuroinflammation relevance because it is the canonical compound used to test whether NLRP3 drives disease phenotypes in CNS models [16,169].	Arguably the most important preclinical anchor in the field; recent screening/development reviews still describe MCC950 as the most used preclinical NLRP3 inhibitor [180,181,182].	No useful mature efficacy trial. Review literature indicates that safety concerns, including liver toxicity in *humans*, limited clinical translation of MCC950 itself [180].	High preclinical/low clinical maturity. Essential as a mechanistic tool, but not the lead translational clinical candidate.
**Therapy—ZYIL1/NT-0796 and related MCC950-derived clinical molecules**			
Mechanism—Next-generation MCC950-derived NLRP3 inhibitors are designed to preserve potency while improving drug-like properties and development potential [10]. NT-series are mainly isopropyl esters. *NT-0796* is cleaved to generate the potent inhibitor NDT-19795. NT-0796 itself is a selective antagonist for NLRP3 inflammasome in CNS that passes BBB, shows promising PK/PD profile with no liver toxicity. However, the cleaved product, NDT-19795 has poor CNS penetration. Another selective antagonist, *NT-0249*, addresses peripheral chronic inflammatory diseases [183], and results regarding safety in *humans* are promising. *NT-0167* is another NLRP3 inflammasome antagonist with demonstrated *human* safety, tolerability, BBB-penetrability and superior pharmacokinetic and pharmacodynamic qualities.			
Neuroinflammation	Preclinical evidence	*Human* trials	Translational maturity
Relevant because the field is actively trying to move from strong mechanistic compounds to safer clinical candidates, including molecules that may eventually be more useful for CNS indication [174,184]	Their rationale is largely inherited from the MCC950 pharmacology platform and broader NLRP3 disease biology [175,184].	A 2023 translational immunology paper notes that ZYIL1 (NCT04972188) and NT-0796 had initiated phase I safety trials [184]. *NT-0249* is currently tested in Phase 1 clinical trials to address peripheral chronic inflammatory diseases. NT-0167 will soon be examined in clinical trials for the treatment of patients suffering from inflammatory and neurodegenerative diseases [165]	Early clinical maturity. Important to mention as the “next wave,” but still early-phase and not yet clinically proven.
**Tranilast**			
Mechanism—Not a clean, modern selective NLRP3 inhibitor; mostly an older or broader inflammasome-modulating strategy [11,185].			
Neuroinflammation	Preclinical evidence	*Human* trials	Translational maturity
Less ideal as selective NLRP3 pharmacology [185]	Mainly review-level support [186].	No strong CNS-focused direct trial signal surfaced here.	Lower priority on selective NLRP3 inhibition.

**Table 3:** Upstream NLRP3 pathway modulators in neuropsychiatric disease.

**MitoQ**			
Mechanism—Mitochondria-targeted antioxidant that accumulates in mitochondria and is intended to reduce mtROS at source [192].			
**Neuroinflammation**	**Preclinical evidence**	***Human* trials**	**Translational maturity**
Strong rationale for limiting ROS-driven microglial activation, inflammasome priming, and oxidative amplification of neuroinflammation [193].	Strong preclinical positioning in mitochondrial dysfunction/neurodegeneration reviews; repeatedly cited as a leading mtROS-directed approach [194,195,196].	PD RCT: Snow et al., [192] tested MitoQ as a disease-modifying therapy in PD; this is the key neurologic *human* trial, but it did not slow progression. Small non-neurologic physiology studies show vascular target engagement in older adults, including improved vascular function and acute endothelial effects [197,198].	Moderate translational maturity. Strong mechanistic and preclinical rationale; *human* safety/physiology signal exists, but neurologic efficacy remains unproven.
**Elamipretide (SS-31)**			
Mechanism—Cardiolipin-binding mitochondrial peptide intended to preserve inner-membrane/cristae integrity, stabilize ETC function, and secondarily reduce ROS generation [199].			
**Neuroinflammation**	**Preclinical evidence**	***Human* trials**	**Translational maturity**
Highly relevant upstream therapy because maintaining membrane integrity may reduce bioenergetic failure, mtROS excess, and release of inflammatory mitochondrial danger signals [199,200].	Strong mechanistic support across mitochondrial disease/neurodegeneration literature; better viewed as a **mitochondrial integrity** therapy than a pure antioxidant [199].	Clinically advanced in primary mitochondrial myopathy. MMPOWER-3 was overall negative in a heterogeneous PMM population; later post hoc work suggested genotype-specific benefit in selected subgroups [201]	Moderate-to-high. Advanced mitochondrial therapeutic platform, but not yet validated as a broad neuroinflammation therapy
**Urolithin A (UA)**			
Mechanism—Mitophagy inducer that promotes the removal of dysfunctional mitochondria and improves mitochondrial quality control [191,202].			
**Neuroinflammation**	**Preclinical evidence**	***Human* trials**	**Translational maturity**
Very strong fit for your framing: by clearing damaged mitochondria, UA may reduce persistent mtROS, mitochondrial DAMP signaling, and inflammatory immune-cell dysfunction [191,202].	One of the most consistently highlighted pharmacologic mitophagy enhancers in neurodegeneration and microglial-mitophagy reviews [191,202].	Phase 3 MMPOWER-3 was negative overall in the heterogeneous PMM population; later post hoc analysis suggested genotype-specific benefit in selected subgroups [191]. Older adults: JAMA Network Open trial showed effects on muscle endurance and mitochondrial health [203]. Immune aging: Nature Aging RCT showed UA as a mitophagy inducer in age-related immune decline, extending its relevance to inflammaging biology.	Moderate-to-high translational maturity. Clinically advanced mitochondrial therapeutic, but not yet validated as a broad anti-neuro-inflammatory or CNS disease-modifying therapy. High translational maturity within the mitophagy field. Best-supported *human* mitophagy inducer, though CNS-specific efficacy trials are still lacking [203].
**Spermidine**			
Mechanism—Autophagy/mitophagy-supporting polyamine; often described as an autophagy inducer with mitophagy-promoting effects [204].			
**Neuroinflammation**	**Preclinical evidence**	***Human* trials**	**Translational maturity**
Relevant to neuro-inflammation through improved mitochondrial turnover and reduced accumulation of damaged, inflammation-driving mitochondria in brain aging [204]	Strong support in aging/neurodegeneration reviews and animal work linking spermidine to improved cognitive/brain aging phenotypes and autophagy-dependent protection [205,206].	Safety/tolerability study in older adults with subjective cognitive decline [207]. Randomized study in older adults with subjective cognitive decline assessed cognition and biomarkers; useful as an early brain-aging translational bridge, but not definitive efficacy proof [208].	Moderate translational maturity. Good early *human* cognitive-aging signal, but evidence is still from small studies and not disease-modifying.
**Dietary antioxidants (generic class)**			
Mechanism—Broad exogenous redox buffering through scavenging ROS and supporting endogenous antioxidant defenses [209,210].			
**Neuroinflammation**	**Preclinical evidence**	***Human* trials**	**Translational maturity**
Relevant because oxidative stress amplifies microglial activation, mitochondrial dysfunction, lipid peroxidation, and neuronal injury in neuro-degeneration [209,211].	Strong general preclinical rationale across AD/PD and related disorders, but the class is heterogeneous and not mechanistically uniform [209].	*Human* evidence is diffuse and inconsistent; benefit is hard to attribute to the class as a whole because formulation, dose, timing, and disease stage vary widely [212,213,214].	Low-to-moderate translational maturity as a class. Biologically plausible, but too heterogeneous to function as a precise therapeutic category.
**N-acetyl-cysteine (NAC)**			
Mechanism—Cysteine donor that replenishes glutathione (GSH) and supports intracellular redox buffering; also used as a cysteine-based antioxidant [215,216].			
**Neuroinflammation**	**Preclinical evidence**	***Human* trials**	**Translational maturity**
Strong relevance as GSH depletion and oxidative stress are linked to mitochondrial dysfunction and inflammatory amplification in PD and other neurodegenerative settings [216,217].	Reviews of antioxidant therapy in PD highlight NAC as one of the more rational antioxidant candidates because it directly supports the GSH system rather than acting only as a free-radical scavenger [216,217].	Small *human* studies in PD. One prospective oral NAC study increased peripheral redox markers but did not clearly increase brain GSH; another more recent study used combined oral + IV NAC over 3 months [216].	Moderate translational maturity. Better mechanistic footing than many supplements, but still limited by small trials and uncertain CNS target engagement.
**Vitamin E (tocopherols)**			
Mechanism—Lipid-phase antioxidant that protects membranes from lipid peroxidation [218]			
**Neuroinflammation**	**Preclinical evidence**	***Human* trials**	**Translational maturity**
Relevant where membrane oxidative damage is central, especially in AD-like oxidative injury [219].	Strong historic oxidative-stress rationale, but preclinical promise has translated inconsistently [218].	One randomized trial reported that 2000 IU/day vitamin E slowed functional decline in mild-to-moderate AD. However, broader antioxidant-trial literature in AD has often failed to show robust cognitive benefit [220].	Moderate translational maturity. Real *human* trial history and one important positive AD signal, but overall efficacy remains mixed/inconsistent.
**Vitamin C (ascorbic acid)**			
Mechanism—Water-soluble antioxidant and electron donor; often discussed as complementary to vitamin E in controlling oxidative stress [221,222].			
**Neuroinflammation**	**Preclinical evidence**	***Human* trials**	**Translational maturity**
Mechanistically relevant as it buffers oxidative stress and may work in tandem with membrane antioxidants [221].	Broad biochemical rationale, but less compelling as a stand-alone neurotherapeutic than NAC or CoQ10 [221,223].	*Human* neurologic evidence is weak as a single-agent therapy. Reviews discuss vitamin C as part of combined antioxidant strategies, without consistently translating into cognition benefit [221,222].	Low translational maturity as monotherapy. Mechanistically plausible but clinically underpowered and not well validated alone.
**Coenzyme Q10**			
Mechanism—Mitochondrial electron carrier and antioxidant that supports OXPHOS and helps reduce oxidative stress [224,225]			
**Neuroinflammation**	**Preclinical evidence**	***Human* trials**	**Translational maturity**
Strong relevance because mitochondrial dysfunction and ROS generation are central to neurodegeneration, particularly PD [224,225]	Preclinical PD literature repeatedly supports CoQ10 as neuroprotective for dopaminergic neurons and mitochondrial function [224].	Several PD trials. Major phase III QE3 trial found no significant benefit on UPDRS progression despite high-dose CoQ10 [224,226]	Moderate translational maturity. Strong mechanistic rationale and substantial trial history, but pivotal efficacy in PD was negative.
**Curcumin**			
Mechanism—Multifunctional antioxidant/polyphenol with effects on ROS, inflammatory signaling, and sometimes NF-κB-related pathways; often limited by poor bioavailability [227,228,229].			
**Neuroinflammation**	**Preclinical evidence**	***Human* trials**	**Translational maturity**
Relevant because curcumin can affect both oxidative stress and inflammatory signaling in preclinical neurodegeneration models. [227,229]	Strong preclinical promise in CNS oxidative injury and neuro-inflammation models, including AD/TBI-related contexts [228].	Curcumin has been tested clinically in AD, but review literature notes no evident clinical efficacy, likely due in part to poor bioavailability [228,230].	Moderate preclinical/low clinical maturity. Attractive biology, weak *human* efficacy so far.
**Resveratrol**			
Mechanism—Polyphenol with antioxidant and signaling effects, often linked to SIRT1, mitochondrial function, and anti-inflammatory pathways [231,232]			
**Neuroinflammation**	**Preclinical evidence**	***Human* trials**	**Translational maturity**
Relevant because it sits at the interface of oxidative stress, mitochondrial homeostasis, and inflammatory signaling [231].	Strong preclinical neuroprotection narrative across neurodegeneration literature [231,233,234].	*Human* neurologic trial evidence exists but remains limited and mixed; this retrieved set supports resveratrol mainly through review-level translational discussions rather than a decisive efficacy trial [235].	Moderate preclinical/low-to-moderate clinical maturity.

**Table 4:** Indirect NLRP3 pathway modulators in neuropsychiatric disease—via metabolic reprogramming. AD—Alzheimer’s disease; AMPK—AMP-activated protein kinase; BHB—β-hydroxybutyrate; DMF—Dimethyl fumarate; GAPDH—Glyceraldehyde-3-phosphate dehydrogenase; KEAP1—Kelch-like ECH-associated protein 1; MCI—Mild cognitive impairment; mTOR—Mechanistic target of rapamycin; mTORC1—Mechanistic target of rapamycin complex 1; NLRP3—NLR family pyrin domain containing 3; NRF2—Nuclear factor erythroid 2-related factor 2.

**Dimethyl Fumarate (DMF)**			
Mechanism—Indirect immunometabolic reprogrammer. DMF modifies KEAP1–NRF2 signaling and also targets GAPDH/aerobic glycolysis, shifting immune cells away from pro-inflammatory metabolic states [237].			
Neuroinflammation	Preclinical evidence	*Human* trials	Translational maturity
Strong relevance because glycolytic, proinflammatory microglia/macrophages support IL-1β-rich inflammasome biology; DMF suppresses inflammatory immune activation without being a selective NLRP3 binder [238].	Key mechanistic anchor: Kornberg et al., [239] showed DMF targets GAPDH and aerobic glycolysis to modulate immunity. In neuro models, DMF improved cognitive impairment and neuroinflammation in AD mice via NRF2-linked mechanisms [240].	Clinically established in multiple sclerosis; large *human* exposure exists, though not as an “inflammasome trial.” Real-world comparative MS effectiveness data are available [241]. There is also an AD-focused clinical trial design/rationale paper, but not yet mature AD efficacy data in this set [242].	High translational maturity as a drug; moderate maturity for the specific inflammasome-by-metabolic-reprogramming framing. Best clinically grounded entry in this table.
**Ketogenic diet/β-hydroxybutyrate (BHB)**			
Mechanism—Metabolic substrate switch from glucose dependence toward ketone oxidation; associated with reduced glycolytic pressure, altered mitochondrial metabolism, AMPK/mTOR/autophagy effects, and, in some contexts, BHB-mediated suppression of inflammatory signaling [243].			
Neuroinflammation	Preclinical evidence	*Human* trials	Translational maturity
Strong conceptual relevance because ketone metabolism can reduce oxidative stress, favor mitochondrial efficiency, and dampen pro-inflammatory immune activation; BHB has been described as an endogenous NLRP3 inhibitor in experimental settings [244]	Yamanashi et al. [74] showed BHB attenuated stress-induced behavioral and inflammatory responses and described BHB as an endogenous NLRP3 inhibitor. Reviews also summarize KD/BHB effects on neuroinflammation and mitochondrial stress in neuro-degenerative conditions [243,244].	*Human* neurologic evidence exists but is still scattered and modest: ketogenic interventions have been explored in PD, MS, and MCI/AD-related cognitive studies, mostly as small dietary or metabolic studies rather than definitive inflammasome trials [243,244].	Moderate translational maturity. Strong biology, real *human* feasibility, but still heterogeneous and not yet a standardized inflammasome-targeting therapy.
**Metformin**			
Mechanism—AMPK activator and indirect mitophagy/immunometabolic modulator; tends to oppose mTOR-driven anabolic inflammatory states and can redirect immune metabolism away from damaging inflammatory programs [245,246].			
Neuroinflammation	Preclinical evidence	*Human* trials	Translational maturity
Relevant because AMPK activation is widely linked to reduced inflammatory tone, better mitochondrial quality control, and lower propensity for persistent inflammasome signaling in activated myeloid cells [245]	Microglial mitophagy review literature explicitly cites metformin as a pharmacologic inducer of mitophagy and links metabolic stress/mtROS to inflammatory microglial polarization [247].	*Human* neurologic use exists in broader repurposing discussions, but there is no strong CNS clinical trial paper specifically tying metformin to inflammasome modulation [187,245].	Moderate mechanistic maturity/low-to-moderate translational specificity. Good biology, but the neuro-inflammasome clinical framing remains indirect.
**Rapamycin/mTOR inhibition**			
Mechanism—mTORC1 suppression, promoting autophagy, altering immune cell biosynthesis/glycolysis programs, and generally shifting cells away from highly inflammatory metabolic states [248,249].			
Neuroinflammation	Preclinical evidence	*Human* trials	Translational maturity
Relevant because excessive mTOR signaling supports inflammatory metabolic programs, while autophagy induction can reduce the persistence of damaged organelles and inflammatory danger signaling [248].	Review literature on microglial mitophagy and neuroinflammation repeatedly cites rapamycin as a compound that can trigger mitophagy/autophagy and support anti-inflammatory reprogramming [248,250].	Limited	Moderate mechanistic maturity/low clinical maturity for this specific question. Strong in discussion sections, weaker as a clinically validated example.

**Table 5:** Upstream inflammatory priming/ER-Golgi and trafficking targets in neuropsychiatric disease. 4-PBA—4-Phenylbutyric acid; AD—Alzheimer’s disease; ATP—Adenosine triphosphate; BTK—Bruton’s tyrosine kinase; CNS—Central nervous system; ER—Endoplasmic reticulum; ISR—Integrated stress response; NLRP3—NLR family pyrin domain containing 3; P2X7—Purinergic receptor P2X ligand-gated ion channel 7; TUDCA—Tauroursodeoxycholic acid; UPR—Unfolded protein response.

**P2X7 Antagonism**			
Mechanism—Blocks ATP-driven upstream danger sensing and K^+^ efflux-linked licensing of inflammasome activation; acts before or at the activation threshold rather than directly binding NLRP3 [252]			
Neuroinflammation	Preclinical evidence	*Human* trials	Translational maturity
Very high relevance in CNS sterile inflammation as extracellular ATP is a classic danger signal in microglia, and P2X7 is a major upstream node for inflammasome-prone neuroinflammatory signaling.	Neurology-focused inflammasome reviews consistently place P2X7 among the best upstream targets in AD/PD/stroke-like neuroinflammatory models.	*Human* clinical development exists in neuropsychiatric and inflammatory contexts historically, but there is no decisive modern neurologic efficacy paper tied specifically to inflammasome suppression.	Moderate translational maturity. Strong mechanistic and CNS relevance, but published disease-modifying neuro data remain limited in this set.
**BTK inhibition**			
Mechanism—Suppresses an upstream signaling kinase that contributes to NLRP3 priming and activation competence in myeloid cells; can reduce inflammasome-supportive signaling without being a direct NLRP3 blocker [253]			
Neuroinflammation	Preclinical evidence	*Human* trials	Translational maturity
High relevance because BTK is expressed in immune compartments and is increasingly discussed as a bridge between innate signaling, microglial activation, and CNS inflammatory pathology [253,254,255].	Targeting the NLRP3 Inflammasome via BTK, frames BTK as an upstream inflammasome-regulatory target. CNS-oriented reviews also discuss BTK inhibition as promising in neurological disorders [253]	Clinically, BTK inhibitors are already established drugs in oncology and are in CNS immunology development, but there is no primary neurologic inflammasome-focused efficacy trial [256].	Moderate translational maturity. Strong druggability and real clinical platform, but inflammasome-specific neurologic validation is still emerging.
**ER-stress/UPR modulators (e.g., TUDCA, 4-PBA, ISR/UPR-targeted strategies)**			
Mechanism—Reduce ER stress, maladaptive UPR signaling (IRE1/PERK/ATF6-related), and proteostasis stress that can feed inflammatory priming and inflammasome-permissive states [257,258].			
Neuroinflammation	Preclinical evidence	*Human* trials	Translational maturity
Strong relevance because chronic ER stress is tightly linked to neurodegeneration, immune activation, and inflammatory amplification in AD and related disorders.	Best mechanistic support comes from reviews on ER stress–NLRP3 interplay and UPR in neuroinflammation, which consistently position the ER–UPR axis as an upstream inflammatory regulator.	*Human* neurologic trials exist for some ER-stress modulators such as TUDCA [259] in broader neurodegeneration, but no inflammasome-specific CNS trial paper.	Moderate mechanistic maturity/low-to-moderate inflammasome-specific clinical maturity. Very useful for mechanistic framing, less mature as a validated inflammasome-target class.

## Data Availability

No new data were created or analyzed in this study.

## Abbreviations

The following abbreviations are used in this manuscript:

AD	Alzheimer’s disease
AIM2 receptors	absent in melanoma 2 receptors
AMPK	AMP-activated protein kinase
ApoE4	apolipoprotein E4
ASC	apoptosis-associated speck-like protein containing a CARD
ASD	autism spectrum disorder
ATP	adenosine triphosphate
Aβ	amyloid-beta
BBB	blood–brain barrier
BD	bipolar disorder
cAMP	cyclic adenosine monophosphate
CAT	catalase
CCL2	C-C Motif Chemokine Ligand 2
CCL20	C-C Motif Chemokine Ligand 2
CLIC	chloride intracellular channels
CNS	central nervous system
CoQ	coenzyme Q10
COX-2	cyclooxygenase 2
CRP	C-reactive protein
CXCL2	C-X-C Motif Chemokine Ligand 2
DAMPs	damage-associated molecular patterns
Drp1	dynamin-related protein 1
ECM	extracellular matrix
ER	endoplasmic reticulum
ETC	electron transport chain
fMRI	Functional Magnetic Resonance Imaging
GABA	gamma-aminobutyric acid
GLT-1	glutamate transporter-1
GMF	glial maturation factor
GSDMD	gasdermin D
GSH	glutathione
HGMB1	high-mobility group box 1
HPA	hypothalamic–pituitary–adrenal
IDO	indoleamine 2,3-dioxygenase
IL-1β	interleukin 1β
IL-18	interleukin 18
IMM	inner mitochondrial membrane
iNOS	inducible nitric oxide synthase
iPSC	induced pluripotent stem cells
LO	lipoxygenase
LRR	leucine-rich repeat
MAM	mitochondria-associated membranes
MAPK	Mitogen-activated protein kinase
MAPKs	mitogen-activated protein kinases
MCP-1	monocyte chemoattractant protein-1
MDD	major depressive disorder
MMPs	metalloproteinases
mtDNA	mitochondrial DNA
mtROS	mitochondrial reactive oxygen species
MyD88	myeloid differentiation primary response 88
NAC	N-acetyl-L-cysteine
NADPH	nicotinamide adenine dinucleotide phosphate
NEK7	NIMA-related kinase 7
NFTs	neurofibrillary tangles
NF-κB	nuclear factor kappa B
NLRC4	NLR family CARD domain-containing protein 4
NLRP1	NLR family pyrin domain-containing protein 1
NLRP3	nucleotide-binding domain, leucine-rich repeat family, pyrin domain-containing protein 3
NMDA	N-methyl-D-aspartate
NO	nitric oxide
NSAID	nonsteroidal anti-inflammatory drugs
NLR	nucleotide-binding domain (NBD)-like receptors
P2X7R	purinergic receptor P2X, type 7
PAMPs	pathogen-associated molecular patterns
PBMC	peripheral blood mononuclear cells
PET	positron emission tomography
PNNs	perineuronal nets
PTSD	post-traumatic stress disorder
RAGE	receptor for advanced glycation end-products
RNS	nitrogen species
RNS	reactive nitrogen species
ROS	reactive oxygen species
SOCE	store-operated Ca^2+^ entry
SOD	superoxide dismutase
SPS	single prolonged stress
SZ	schizophrenia
TFs	tocopherols
TJs	tight junctions
TLR	Toll-like Receptor
TLR4	Toll-like receptor 4
TLRs	Toll-like receptors
TNF-α	tumor necrosis factor alpha
Treg	regulatory T cells
TRX	thioredoxin
TSPO	18 kDa Translocator Protein
TTs	tocotrienols
TXNIP	Thioredoxin-interacting protein
VDAC1	voltage-dependent anion channel 1
YKL-40	chitinase-3-like protein 1
ZO-1	zonula occludens
